# Greater cane rat algorithm (GCRA): A nature-inspired metaheuristic for optimization problems

**DOI:** 10.1016/j.heliyon.2024.e31629

**Published:** 2024-05-23

**Authors:** Jeffrey O. Agushaka, Absalom E. Ezugwu, Apu K. Saha, Jayanta Pal, Laith Abualigah, Seyedali Mirjalili

**Affiliations:** a Department of Computer Science, Federal University of Lafia, Lafia 950101, Nigeria; b Unit for Data Science and Computing, North-West University, 11 Hoffman Street, Potchefstroom, 2520, South Africa; c Department of Mathematics, National Institute of Technology Agartala, Agartala, Tripura, 799046, India; d Department of IT, Tripura University, Suryamaninagar, Tripura 799022, India; e Hourani Center for Applied Scientific Research, Al-Ahliyya Amman University, Amman 19328, Jordan; f Computer Science Department, Al Al-Bayt University, Mafraq 25113, Jordan; g MEU Research Unit, Middle East University, Amman, Jordan; h Applied science research center, Applied science private university, Amman 11931, Jordan; i Centre for Artificial Intelligence Research and Optimization, Torrens University, Australia

**Keywords:** Greater cane rat algorithm, optimization, metaheuristic, population-based, nature-inspired, CEC 2011, CEC 2020, real-world problem

## Abstract

This paper introduces a new metaheuristic technique known as the Greater Cane Rat Algorithm (GCRA) for addressing optimization problems. The optimization process of GCRA is inspired by the intelligent foraging behaviors of greater cane rats during and off mating season. Being highly nocturnal, they are intelligible enough to leave trails as they forage through reeds and grass. Such trails would subsequently lead to food and water sources and shelter. The exploration phase is achieved when they leave the different shelters scattered around their territory to forage and leave trails. It is presumed that the alpha male maintains knowledge about these routes, and as a result, other rats modify their location according to this information. Also, the males are aware of the breeding season and separate themselves from the group. The assumption is that once the group is separated during this season, the foraging activities are concentrated within areas of abundant food sources, which aids the exploitation. Hence, the smart foraging paths and behaviors during the mating season are mathematically represented to realize the design of the GCR algorithm and carry out the optimization tasks. The performance of GCRA is tested using twenty-two classical benchmark functions, ten CEC 2020 complex functions, and the CEC 2011 real-world continuous benchmark problems. To further test the performance of the proposed algorithm, six classic problems in the engineering domain were used. Furthermore, a thorough analysis of computational and convergence results is presented to shed light on the efficacy and stability levels of GCRA. The statistical significance of the results is compared with ten state-of-the-art algorithms using Friedman's and Wilcoxon's signed rank tests. These findings show that GCRA produced optimal or nearly optimal solutions and evaded the trap of local minima, distinguishing it from the rival optimization algorithms employed to tackle similar problems. The GCRA optimizer source code is publicly available at: https://www.mathworks.com/matlabcentral/fileexchange/165241-greater-cane-rat-algorithm-gcra.

## 1 Introduction

Optimization is a ubiquitous process, integral to every human endeavor. It involves finding the most effective combination of constraints or decision variables within a defined boundary to solve a specific problem. The challenge of finding optimal solutions across various domains necessitates the use of optimization methods [1]. As such, there is a common need to develop an optimization algorithm that can handle the complexity of scientific and engineering problems. While there is no dearth of optimization methods in the literature [2,3], each has its strengths and weaknesses, ranging from traditional optimization methods to more recent population-based, nature-inspired metaheuristic algorithms.

Traditional optimization methods include linear and non-linear programming techniques [4]. Although these methods are highly effective in solving optimization problems, they are gradient-dependent and can be significantly influenced by the nature and diversity of the initial population [[5], [6], [7], [8]]. Conversely, newer population-based, nature-inspired metaheuristic algorithms have proven to be effective optimization tools for certain optimization problems. However, they do not guarantee optimal solutions for various optimization problems [9] and are prone to getting trapped in local (suboptimal) solutions [10].

Interestingly, optimization problems in different real-world domains come with varying complexities and challenges. They could be linear or non-linear, convex or non-convex, complicated and constrained objective functions, and involve many decision variables. Further, these problems have varying local and global optimums [1]. Solving these problems involves starting from a decent point and navigating the search space to arrive at a global solution. Many methods that attempt to solve these problems exist in the literature and are usually classified into exact and approximate methods [2].

Exact methods guarantee that an optimal solution would be found in polynomial time, provided the problem is not an NP problem. Conversely, the approximate methods do not guarantee optimal solutions but near-optimal ones in polynomial time. The performance of these methods is measured by how close the found solutions are to the global optimal [3]. The past decades have witnessed significant interest in approximate methods, which consist of traditional and heuristic or metaheuristic optimization methods.

Traditional optimization methods effectively handle simple engineering problems with linear search space and less complexity [4]. The performance of these methods depends heavily on knowledge of the problem. It often finds only local solutions (trapped in local optima) for complex problems with many constraints and non-linear problem space [5]. Furthermore, these solutions must be found within a reasonable time.

The difficulty with these conventional optimization techniques is that multiple optimal solutions often exist for most real-world optimization problems. As such, the traditional methods are ineffective for these problems because they rely on the initial solution (which may be suboptimal) around which the global solution is searched [6]. Metaheuristic optimization offers a way out of these challenges, and researchers have shifted their attention toward this optimization method [7,8].

### 1.1 Metaheuristic algorithms

In the context of computer science and mathematical optimization, metaheuristic optimization is a superior-level process or heuristic designed to locate an adequately satisfactory or approximate solution to an optimization problem [9]. There are three basic ways of developing such optimization methods: improvement of existing optimizers, hybridizing two or more existing optimizers, and designing entirely new optimizers. Additionally, numerous classifications of this optimization technique are present in scholarly works, such as population-based versus single-solution, and nature-inspired versus non-nature-inspired, among others [4].

A classification based on a source of inspiration infers that the processes of a natural phenomenon are used to carry out the optimization process. The commonly used phenomenon can be summarized as either mimicking some biological behavior, way of life of some living things, human activity, or physical phenomenon [10]. A summary of this classification is given as follows.

#### 1.1.1 Evolutionary-based algorithms (EAs)

The EAs are the oldest and one of the common metaheuristic algorithms whose source of inspiration is the theory of evolution relying on the survival of the fittest. The optimization process begins with an initial population, randomly generated, that continuously evolves into the subsequent generation, eliminating the least effective solutions in the process. The algorithms in this category generally have the advantage of finding optimal solutions or solutions very close to the optimal solution. Some examples of algorithms in this category can be found in Ref. [4]. However, some of the algorithms published within the last decade in this category are listed in Table 1.

**Table 1:** Algorithm listing for EAs.

Algorithm	Source of Inspiration	Application area	Year
Evolutionary Mating Algorithm (EMA) [11]	The concept of random mating from the Hardy-Weinberg equilibrium, along with the crossover index, is utilized to generate new offspring.	Optimal Power Flow (OPF) problems	2022
Methylation evolution-based bi-gram (MethEvo) [12]	Evolutionary-based bi-gram	Post Translational Modification (PTM)	2022
Predictor-assisted evolutionary neural architecture Search (PRE-NAS) [13]	Strategies for evolutionary search are incorporated, along with the integration of high-fidelity weight inheritance across multiple generations.	NAS-Bench-201 and DARTS search spaces	2022
Heuristic initialization based modified ACO (HIMACO) [14]	Ant safety features	Multicast routing and its parameter tuning	2022
An online generation-less genetic algorithm (OLGGA)	continuous changes in organisms	knapsack optimization problem	2022
Synergistic fibroblast optimization (SFO) [15]	Migration and methodical behavior of the fibroblast	Non-linear complicated optimization problem	2022
Corona virus optimization [16]	Corona virus pandemic	Benchmark functions	2022
Learner Performance-based Behavior (LPB) [17]	Studying behaviors of learners in a university	CEC-C06 2019 test functions and generalized assignment problem (GAP)	2021
Physarum-inspired computational model [18]	Physarum-inspired model.	The traveling salesman problem (TSP)	2019
Stochastic Fractal Search (SFS) [19]	Natural phenomenon of growth	Engineering design optimization problems	2015
Backtracking Search Algorithm (BSA) [20]	Evolutionary concepts with memory	Numerical optimization problems	2013

#### 1.1.2 Swarm intelligence-based (SI) algorithms

This category mimics the dynamic collative intelligence and behaviours of groups, flocks, herds, or communities of creatures in their natural habitat. Community behaviours are observed in the flock of birds, fish schools, colonies of insects, herds of animals, and many more [21]. The group members are independent but cooperate to achieve their collective goal, foraging or migration. Cooperation is emulated to address intricate optimization challenges. This category stands as the most extensive among all, with comprehensive examples available in Ref. [22]. Some recent SI algorithms are presented in Table 2.

**Table 2:** Algorithm listing for SIs.

Algorithm	Source of Inspiration	Application area	Year
Slime mould algorithm [23]	Oscillation mode of slime mould	Classical engineering structure problems	2020
Border collie optimization [24]	Herding style of the Border Collie dog	Benchmark functions	2020
Sparrow Search Algorithm [25]	Group wisdom, foraging, and anti-predation behaviours of sparrows	Benchmark function and engineering problems	2020
Capuchin Search Algorithm [26]	Dynamic behavior of capuchin monkeys	Benchmark function and engineering problems	2021
Chameleon Swarm Algorithm	Dynamic behavior of chameleons	Constrained and computationally expensive engineering design problems	2021
Aquila Optimizer [8]	Aquila's behaviors	CEC2017, CEC2019 test functions, and engineering problems.	2021
Ebola Optimization Search Algorithm [27]	Propagation mechanism of the Ebola virus	Image classification of digital mammography	2021
Rock Hyraxes Swarm Optimization [28]	Collective behavior of Rock Hyraxes	Benchmark functions	2021
Red Colobuses Monkey [29]	Behavior related to red monkeys	Benchmark functions	2021
African vultures optimization algorithm [30]	African vultures' lifestyle	Engineering problems	2021
Battle royale optimization algorithm [31]	Digital games knowns as “battle royale.”	The inverse kinematics problem of the 6-DOF PUMA 560 robot arm	2021
Artificial Jellyfish Search [32]	Jellyfish behavior	Benchmark functions and optimization problems.	2021
Poplar Optimization Algorithm [33]	Sexual and asexual propagation mechanism of poplar	Image segmentation	2022
Snake Optimizer [34]	Special mating behavior of snakes	Constrained real-world engineering problems	2022
War Strategy Optimization [35]	Strategic movement of army troops during the war	Engineering models	2022
Honey Badger algorithm [36]	Intelligent foraging behavior of honey badger	Engineering design problems and CEC’17 test suite.	2022
Gannet optimization algorithm [37]	Unique behaviors of gannets during foraging	Engineering optimization problems	2022
Trees Social Relations Optimization Algorithm [38]	Hierarchical and collective life of trees in the jungle	Continuous and discrete optimization problems	2022
Reptile Search Algorithm [39]	Hunting behaviour of Reptiles	Classical, CEC2017, CEC2019 test functions and engineering problems.	2022
Prairie dog optimization [7]	Foraging behavior of prairie dogs	Engineering optimization problems	2022
Gazelle Optimization Algorithm [40]	Survival adaptation of gazelles	Engineering problems	2022
Dwarf mongoose optimization algorithm [41]	Foraging behavior of dwarf mongoose	Benchmark and engineering problems	2022

#### 1.1.3 Human-based algorithms (HAs)

The algorithms in this category mimic phenomena related to human community or behavior, such as teaching or learning, thinking (mathematics), and other human-related activities [42]. The number of algorithms in this category is not as many as in other categories, but lately, it has received its fair share of attention. Several algorithms within this category can be found in Table 3.

**Table 3:** Algorithm listing for HAs.

Algorithm	Source of Inspiration	Application area	Year
Gaining Sharing Knowledge-based Algorithm [43]	The procedure of acquiring and disseminating knowledge throughout human life.	CEC2017 benchmark and IEEE-CEC2011 real-world optimization problems	2020
Coronavirus Herd Immunity Optimizer [44]	Herd immunity concept as a way to tackle the corona virus pandemic (COVID-19)	Engineering optimization problems extracted from IEEE-CEC 2011	2021
The arithmetic optimization algorithm [45]	Arithmetic operators	Engineering application	2021
Ali Baba and the forty thieves [10]	Story of Ali Baba and the forty thieves	Engineering design problems	2022
Driving Training-Based Optimization [46]	Human activity of driving training	IEEE CEC2017 test functions	2022
Human felicity algorithm [47]	Efforts of human society to become felicity	CEC 2014, CEC 2019, CEC2020, and complex engineering problems	2022
Stock exchange trading optimization algorithm [48]	Behavior of traders and stock price changes in the stock market	Engineering design problems	2022
Puzzle Optimization Algorithm [49]	Process of solving a puzzle	Benchmark functions	2022
Child Drawing Development Optimization Algorithm [50]	Based on Child's Cognitive Development	Benchmark functions	2022

#### 1.1.4 Physics-based (PB) algorithms

The algorithms in the classification derive their inspiration from physical phenomena such as physics, chemistry laws, and other physical processes [51]. The optimization mimics the rule governing the physical processes in nature. Some examples of algorithms in this category can be found in Refs. [51–53]. However, a list of recent algorithms in this group is given in Table 4.

**Table 4:** Algorithm listing for PBs.

Algorithm	Source of Inspiration	Application area	Year
Henry gas solubility optimization [52]	The behavior of Henry's law	Engineering design problems and CEC’17 test suite problems.	2019
Equilibrium optimizer [53]	Mass balance models	Mathematical and engineering benchmarks	2020
Archimedes optimization algorithm [54]	Law of physics Archimedes' Principle	Engineering design problems	2021
Lichtenberg Algorithm [55]	Lichtenberg figure pattern	Benchmark functions and design problems in a welded beam	2021
Heat transfer relation-based optimization algorithm [56]	Heat transfer relationships based on the second law of thermodynamics	PID controller and linear regression	2021
The String Theory Algorithm [57]	String Theory,	Optimal design of a fuzzy controller	2021
Crystal Structure Algorithm [58]	Principles underlying the formation of crystal structures	239 mathematical functions	2021

#### 1.1.5 Hybrid based

Hybrid algorithms merge characteristics from multiple optimization algorithms, leading to an enhanced algorithm that optimizes more effectively while simultaneously reducing the computational intricacies of the resulting hybrid [59]. This category of algorithms has the most extensive collection of articles because more research efforts are directed toward this area. Hybrid algorithms make up a long list of algorithms and their variants.

Significant efforts have also been made in applying these modified algorithms in the area of optimal power flow problems to improve load flows and focus on minimizing the objective functions [60]. Additionally, the optimal power flow (OPF) problem of a power system was addressed using a salp swarm algorithm (SSA), which included the integration of the thyristor-controlled series capacitor (TCSC) [61].

### 1.2 Exploration and exploitation

The commonality between all metaheuristic algorithms is that they all aim to find the best possible solution for any optimization problem using two critical transitional phases: exploration and exploitation. The process of optimization with population-based metaheuristic algorithms initiates by randomly producing an initial set of solutions or populations within the defined boundary constraint. The search agents move globally as extensively as possible, looking the better solutions. This global movement is called exploration [62]. The objective of this stage is to pinpoint potential areas within the search space where the universal solution might be located, thus preventing the algorithm from becoming stuck in the local or present solution.

After identifying promising regions, the algorithm must search these areas for the global solution and move towards that solution, thereby improving the quality of solutions found so far. This local movement is called exploitation. The primary aim of this phase is to better the solutions obtained during the exploration phase [25]. Local movement or exploitation also helps avoidance of local optima; however, only local solutions very close to the global optima are avoided. Also, the area covered by the exploitation phase is very small compared to the areas covered by the exploration [63].

The problem most metaheuristic algorithms face is knowing which phase to start and when to transition between the phases [64]. The nature or shape of the problem landscape is unknown, so the timings must be such that the stochastic nature of the algorithms is maintained. As a solution, most algorithms follow the continuous and repeated transition between the phases, trying to maintain a search balance [65]. This solution is implemented in three ways.

• First, the existing algorithm may be modified by parameter tunning or enhancing the optimization processes. The algorithm parameters can influence the quality of the results. It is impossible to know beforehand the best parameter settings. Also, the problem's nature to be solved influences the parameter setting. Therefore, there is no universally optimal parameter setting.
• Secondly, the a need for further improvement of metaheuristics called hybridization. The hybrid algorithms benefit from the synergy between the candidate algorithms for the hybridization. Therefore, which algorithm can complement the other is the key to successful hybridization.

The complexity of optimization problems greatly influences the need to design a new metaheuristic algorithm. Generally, a problem that can be solved in a polynomial time is called easy or tractable otherwise, it is difficult or intractable. Most real-world problems are NP-hard. No universally demonstrably efficient algorithms exist to solve them in polynomial time except in exponential time. Therefore, developing a new metaheuristic algorithm may be a valuable way to solve these NP-hard problems [66].

### 1.3 Motivation

A metaheuristic is commonly judged by its ability and competitiveness in finding solutions to optimization problems. While there is no shortage of algorithms with proven capacity to find optimal solutions to a given optimization problem, there is a wide range of problems yet to be solved by the algorithms, and there is no guarantee that the existing ones would be effective in solving those problems. The ‘‘no-free-lunch’’ (NFL) theory [67] clarifies that one metaheuristic algorithm can solve all optimization problems. The implication is that fine-tuning an algorithm to effectively find optimal solutions to a problem could potentially offset the algorithm's performance, given another problem. This theory could potentially mean fine-tuning or developing new algorithms for every optimization problem. It also keeps the field of metaheuristic optimization open and pushes researchers to endless limits to improve the quality of solutions found to cater to emerging, complex real-world problems.

Beyond the aforementioned points, the subsequent reasons are provided for the creation of the novel GCR metaheuristic algorithm to address the chosen problems that are examined and showcased in this research.

• The nature and adaptation of the greater cane rats are exciting phenomena that have never been used to model any optimization method. The combination of intelligible activities that results in effective foraging during and off mating season provides tools that can be modeled to enhance optimization.
• These activities are unique to the greater cane rats and effectively translate to the exploration and exploitation activities discussed in Section 2.
• The exchange of information about trails during foraging is uniquely modeled by attractive movements of the other GCR towards the position of the dominant rat instead of just directly copying the position from the dominant rat, as seen in Equation (3). This strategy explores regions near the promising region represented by the dominant rat position.
• The reason for using μ,α,β values, as shown in Equations 7, 8, 9, is to maintain diversity and enhance the intensification.
• The selection of the individual GCR follows the tournament approach where the fittest one (according to fitness function) is better between two feasible solutions. A feasible solution is always better than an infeasible one, and between two infeasible solutions, the one having the smaller sum of constraint violations is preferred.

Similarly, the aforementioned reasons are the primary motivation for this work.

### 1.4 Contributions

This study introduces a novel optimization algorithm, the Greater Cane Rat Algorithm (GCRA), which draws inspiration from the discerning foraging behavior of the Greater Cane Rat during and outside the mating season. The GCRA has been employed to tackle both constrained and unconstrained CEC 2011 real-world optimization problems. Furthermore, the GCRA provides an improved balance between exploration and exploitation, leading to the anticipation of superior or more competitive results compared to existing solutions. The primary contributions of this research are outlined below.

• A new optimization algorithm, inspired by the GCR's intelligible foraging behavior during and off mating season new meta-heuristic
• The performance of the proposed GCRA was assessed on 23 classical benchmark functions, 10 CEC 2020 complex functions, 22 real-world optimization problems found in the CEC 2011 suite, and six (6) classical engineering problems.
• The performance of GCRA was compared with other metaheuristics that have been used to solve these problems.

### 1.5 Advantages of GCRA

The proposed GCRA offers several advantages as a tool for global optimization, which are listed as follows.

• The GCRA is flexible, evident in only one parameter to be tuned (representing whether it is a mating season or not). This flexibility makes GCRA adaptable to different optimization problems.
• The proposed mathematical model for GCRA makes it an effective optimization tool for the selected problems in six (6) engineering design problems and the CEC 2011 test suite.
• The GCRA's straightforwardness and resilience enable it to swiftly and precisely identify global solutions with a high rate of convergence.
• Finally, the GCRA is a low-cost and potent tool for challenging real-world optimization.

The rest of this paper is organized as follows: Section 2 presents the proposed algorithm's source of inspiration, and the GCRA's mathematical models are presented in Section 3. The experimental setup, classical benchmark functions, CEC 2020, CEC 2011 problems, six (6) engineering design problems, results, and discussion are presented in Section 4. Finally, the conclusions and future trends are given in Section 5.

## 2 Inspiration

**Fig. 1:**
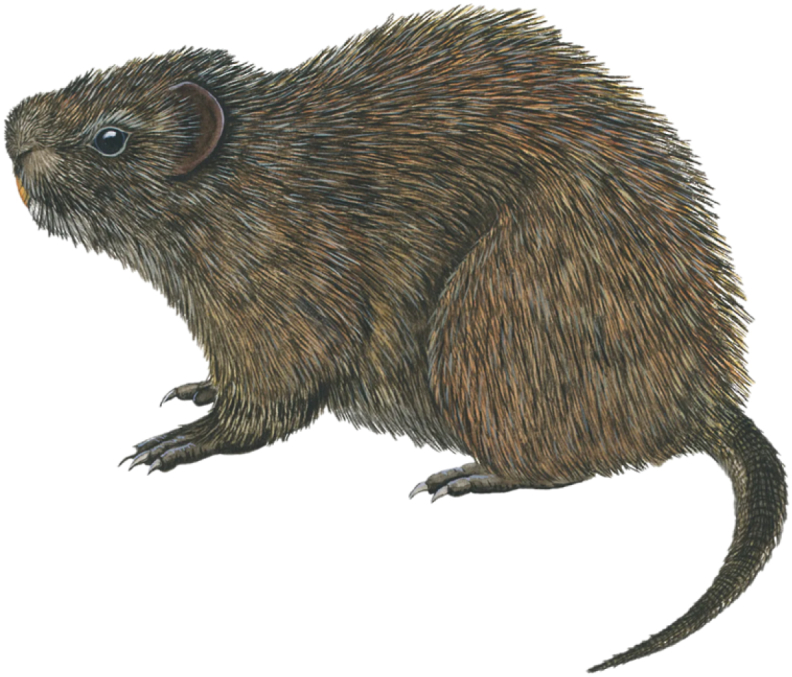
Greater cane rat (image drawn using InkScape version 1.2.1: https://inkscape.org/).

The greater cane rat (Thryonomys swinderianus) is also called the grasscutter in Ghana, Nigeria, and other regions of West Africa. It belongs to the family of cane rats or the African hystricognath rodents. The other member is the lesser cane rat (T. gregorianus) [68]. They live by reed beds, riverbanks, lakes, swamps, and tall, thick cane-like grasses in Sub-Saharan Africa [69]. The greater cane rats are one of the largest rodents in Africa, measuring between 43 and 60 cm head-body, with the tail reaching between 16 and 19.5 cm. The males and females differ in weight, with the males averaging 4.5 kg and the females averaging 3.4–3.8 kg. Generally, they sometimes weigh up to approximately 7–9 kg. Their ears are rounded, coarse-looking, bristly hair, and they have short noses. The front feet are smaller than the hind feet, each with three toes [70]. Fig. 1 shows the GCR, depicting all the features mentioned earlier.

### 2.1 Behavioral adaptations

**Fig. 2:**
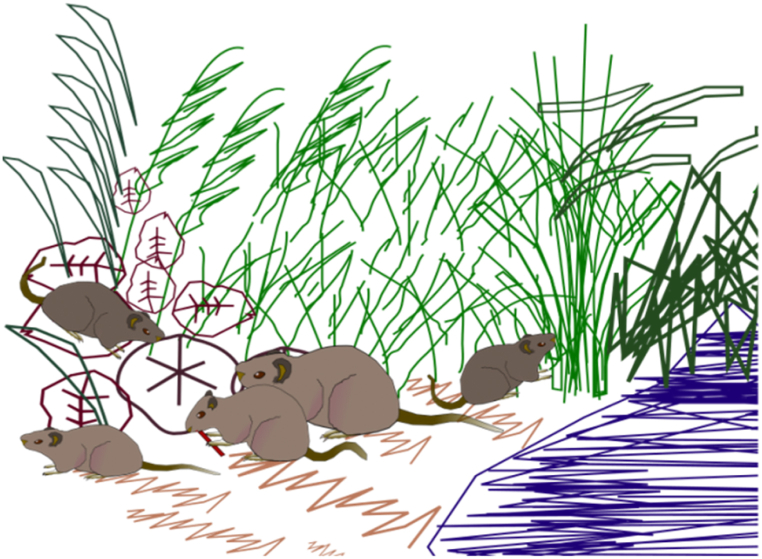
The natural habitat of the GCR (image drawn using InkScape version 1.2.1: https://inkscape.org/).

Greater cane rats are excellent swimmers and use the water as a haven from danger. Also, they are swift and agile on land. They are primarily nocturnal and occasionally active during the day [69]. They are patriarchal and live in a small family group led by a dominant male. Their den is usually a nest in thick vegetation, although they sometimes use underground burrows abandoned by other animals or termites. When danger is sensed, they either grunt or dash for the water [71]. The grass is the major diet of the greater cane rats, though they eat other plants, fruits, and tree bark. As seen in Fig. 2, the GCRs live near a water source depicted by the shaded portion at the base of the figure, tall cane-like grasses are visibly depicted, and the white spaces and paths represent the trails to previously known food sources through the cane-like features.

### 2.2 Assumptions

The greater cane rats (GCR) can be territorial, with cases of fighting between only the males using the nose for duels. They are also social animals living in groups with a dominant male, many females, and juveniles (can be more than a generation old). Greater cane rats' foraging comprises cutting canes and grasses using their specially adapted upper incisors. Being highly nocturnal, they are intelligible enough to leave trails as they forage through reeds and grass. These trails would subsequently lead to food and water sources and shelter [72].

## 3 GCRA model

This section presents the mathematical models and formulations of the proposed GCRA optimization procedures.

### 3.1 Population initialization

The optimization process of GCRA begins with the stochastic generation of the Greater Cane Rats (GCR) population (X) using Equation (1). This generation utilizes the upper bound (UB) and lower bound (LB).

1 $$X=\left[\begin{array}{c} \begin{array}{ccc} x_{1,1} & \begin{array}{cc} x_{1,2} & \cdots \end{array} & \begin{array}{cc} x_{1,d-1} & x_{1,d} \end{array} \end{array}\\ \begin{array}{ccc} x_{2,1} & \begin{array}{cc} x_{2,2} & \cdots \end{array} & \begin{array}{cc} x_{2,d-1} & x_{2,d} \end{array} \end{array}\\ \begin{array}{c} \begin{array}{ccc} \vdots & \;\begin{array}{cc} \vdots & x_{i,j} \end{array}\; & \;\begin{array}{cc} \vdots & \vdots \end{array} \end{array}\\ \begin{array}{ccc} x_{n,1} & \begin{array}{cc} x_{n,2} & \cdots \end{array} & \begin{array}{cc} x_{n,d-1} & x_{n,d} \end{array} \end{array} \end{array} \end{array}\right]$$

where X represents the entire GCR population and the individual rat ($x_{i,j}$) in the ith position in the jth dimension is generated randomly using Equation (2). Finally, n and d denote the population size and the problem dimension, respectively.

2 $$x_{i,j}=\text{rand}\times \left({\text{UB}}_{j}-{\text{LB}}_{j}\right)+{\text{LB}}_{j}$$

where rand is a random number between 0 and 1.

### 3.2 The GCRA model

Greater cane rats (GCR) exhibit territorial behavior, often engaging in duels using their noses, primarily among males. These creatures are social, typically forming groups composed of a dominant male, multiple females, and juveniles that may span more than one generation. Their foraging habits involve cutting canes and grasses with their uniquely adapted upper incisors. As predominantly nocturnal animals, GCRs are adept at leaving trails during their foraging activities through reeds and grass, leading to sources of food, water, and shelter. This intelligent behavior of the GCRs is the central focus of this study. The exploration phase is marked by their departure from various shelters within their territory to forage and create trails.

It is hypothesized that the dominant male retains information about these trails, allowing other rats to update their positions accordingly. Males also have the ability to recognize the breeding season and segregate themselves from the group. It is assumed that during this separation, foraging activities are concentrated in areas with plentiful food sources, facilitating exploitation. This study mathematically models the intelligent foraging trails and actions during the mating season to design the GCR algorithm and carry out optimization activities.

**Fig. 3:**
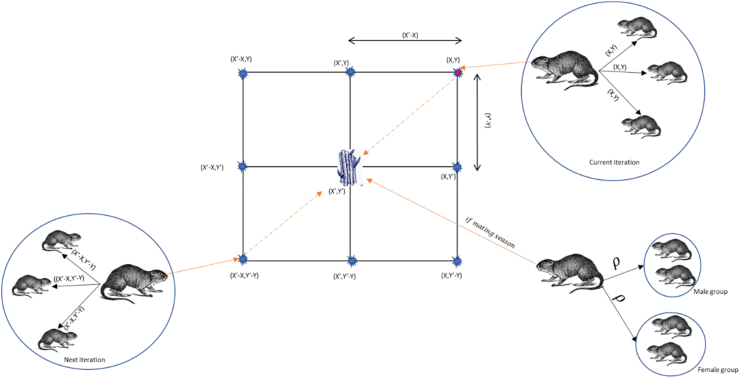
2D possible position vectors (image drawn using InkScape version 1.2.1: https://inkscape.org/).

The conceptual model of the GCR is shown in Fig. 3. The target food source is assumed to be at position (X′, Y′). The path to this food source is known to the alpha male, who then passes this information to the rest of the family members and updates their position based on the information passed. An alpha at position (X, Y) is aware of the food source at position (X′, Y′) and the different neighbouring positions that can be reached depending on the influence of Equations 6, 7). At another iteration, an alpha at position (X′-X, Y′-Y) undergoes the same process. During the mating season, information about the path leading to the plentiful food source is shared. This prompts the family to divide into groups based on gender, with both male and female groups relocating to the area of the food source to establish their camp.

Within the framework of the GCRA design, the rat that is considered dominant is presumed to be the fittest, or the position vector yields the best value of the objective function. Given that this fittest rat guides the group and possesses knowledge of past routes to food or shelter, the positions of the other rats are adjusted according to the location of the dominant male, as per Equation (3). The dominant rat position is denoted by $X_{k}$. The GCRA goes into either the exploration or exploitation phase depending on the value of ρ, which is a variable that determines whether it is a rainy season or not. The value of ρ is carefully selected to balance exploration and exploitation. The value of ρ is carefully tuned to 0.5 after rigorous parametric analysis.

3 $$x_{i,j}^{\text{new}}=0.7\;*\frac{\left(x_{i,j}+x_{k,j}\right)}{2}$$

where $x_{i,j}^{\text{new}}$ denotes the new GCR position, $x_{i,j}$ denotes the current GCR position, $x_{k,j}$ is the dominant male in the jth dimension.

### 3.3 Exploration

**Fig. 4:**
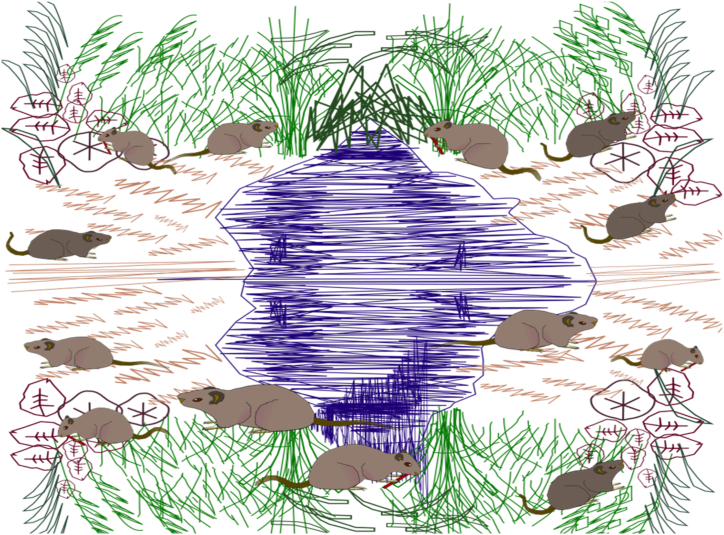
Greater cane rats looking for food sources (image drawn using InkScape version 1.2.1: https://inkscape.org/).

The GCR build their shelter (nest or shallow burrows) scattered around their territory (marshes, riverbanks, and cultivated crop farms). They leave the different shelters to forage by either following trails to previous food sources or scouring for a new food source and leaving trails. Fig. 4 shows the greater cane rats scouring for food everywhere within their territory, the rats that appear to be walking represent the dominant rat's different position, and the rats that appear to be eating represent found food sources. It is assumed that the dominant male retains the information about these trails, and other rats adjust their position based on this data. A new position for the remaining rat population in the search space is determined according to the dominant male's position, as depicted in Equation (4). In this phase of the GCR motion simulation, if another rat's objective function value surpasses that of the fittest rat, the fittest rat is updated, and the positions of the other rats are adjusted based on the newly established fittest rat. If not, it deviates from the fittest rat's position. This GCR movement strategy is modeled in Equation (5). The final step of the exploration phase suggests that the GCR only relocates to this newly calculated position if the objective function value improves at this new location; otherwise, the GCR maintains its previous position.

4 $$x_{i,j}^{\text{new}}=x_{i,j}+C\times \left(x_{k,j}-r\times x_{i,j}\right)$$

5 $$X_{i}=\left\{ \begin{array}{c} x_{i,j}+C\times \left(x_{i,j}-\alpha \times x_{k,j}\right),F_{i}^{\text{new}}<F_{i}\\ x_{i,j}+C\times \left(x_{m,j}-{\beta \times x}_{k,j}\right),\text{otherwise} \end{array}\right.$$

where $X_{i}$ signifies the upcoming or new state of the ith GCR, $x_{i,j}^{\text{new}}$ represents its value in the jth dimension, $x_{i,j}$ indicates the current GCR position, $x_{k,j}$ is the dominant male in the jth dimension, $F_{x_{k}}$ is the value of the dominant male's objective function, $F_{x_{i}}$ is the current value of the objective function, C is a random number defined within the problem space boundaries, simulating the dispersed food sources and shelter, r simulates the effect of an abundant food source, which then prompts more exploitation and is defined in Equation (6), α is a coefficient that simulates a diminishing food source, which compels the search for new food sources or shelter and is defined in Equation (7), β is the coefficient that prompts the GCR to move to other available abundant food sources within the breeding area and is defined in Equation (8). The parameters r,αandβ are modified from concepts in Ref. [73].

6 $$r=F_{x_{k}}-C_{\text{iter}}\times \left(\frac{F_{x_{k}}}{\text{Ma}x_{\text{iter}}}\right)$$

7 α=2×r×rand−r

8 β=2×r×μ−r

where $C_{\text{iter}}$ is the current iteration, ${\mathrm{Max}}_{\text{iter}}$ is the maximum iteration

### 3.4 Exploitation

**Fig. 5:**
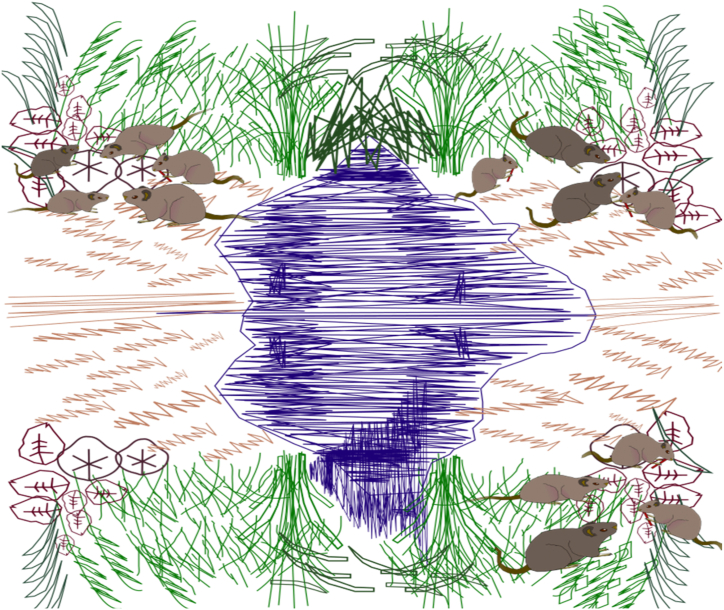
Foraging during mating season (image drawn using InkScape version 1.2.1: https://inkscape.org/).

The breeding season varies from habitat to habitat and usually occurs during the wet season. The males are known to separate themselves from the group during the breeding season. The assumption is that once the group is separated, the foraging activities are concentrated within areas with abundant food sources. Fig. 5 shows the separate groups foraging within the promising regions only. The simulation of the phase starts by randomly selecting a female m such that m≠k (the dominant male). Because breeding occurs around abundant food sources, the intensification occurs around the selected female. The process's modeling is depicted in Equation (9). If the newly computed position for the GCR enhances the target function's value, as modeled in Equation (5), it supersedes the prior position.

9 $$x_{i,j}^{\text{new}}=x_{i,j}+C\times \left(x_{k,j}-\mu \times x_{m,j}\right)$$

where $x_{m,j}$ represents the position of the randomly selected female in the jth dimension, μ randomly takes the values from 1 to 4, simulating the number of young produced by each female GCR per year. The parameters C,r,μ,ρ,α,β are responsible for better exploration and exploitation throughout the iterations.

The pseudocode for the algorithm is given below.

Algorithm 1

**Table:** 

**Parameters**: ρ:
**Input**: greater cane rats’ population, maximum iteration, ρ:
**Output**: optimal search result
**Process**: GCRA optimization
Calculate the fitness of each GCR
Select the fittest GCR as the dominant male $X_{k}$:
Update the global best solution (Gbest)
Update the remaining GCR based on the position of $X_{k}$ using Equation 3
**For** iter = 1: max_iter
Evaluate C,r,μ,α,β:
**If**rand<ρ:
**Exploration**:
Update current search agent position based on Equation (4)
Check boundary constraints
**Else**
**Exploitation**:
Update current search agent position based on Equation (9)
Check boundary constraints
**End If**
Evaluate the fitness of each GCR based on a new position
Update search agent based on Equation (5)
Update Gbest
Select a new dominant male $X_{k}$:
**End For**
**Return** Gbest
**End**

### 3.5 Computational complexity

The Big-O notation is used to express the computational complexity of the proposed algorithm. The computational complexity is the relationship between the input, input size, process, and time it takes to bring out the output. This computational complexity is presented in terms of both space and time.

#### 3.5.1 Time complexity

The time complexity is measured in terms of the number of greater cane rats (n), the problem's dimensions (d), the maximum number of iterations ($\text{Ma}x_{\text{iter}}$) and the number of function evaluations (fe). Therefore, the time complexity of GCRA is given as follows:

10 O(GCRA)=O(problemdef)+O(solcreation)+O(fe)+O(updatingsol)

Equation (10) gives the components of the time complexity of GCRA. The details demand of the components are given as follows.

• Problem definition requires O(1) time.
• The GCRA requires O(n×d) time to create the random population.
• The function evaluations require $O\left({\mathrm{Max}}_{\text{iter}}\times \text{fe}\times n\right)$ time.
• The time required for solution update is $O\left({\mathrm{Max}}_{\text{iter}}\times n\times d\right)$.

Substituting the details of each component in Equation (11) leads to the general time complexity given in Equation (11).

11 $$O\left(\text{GCRA}\right)=O\left(1+\left(n\times d\right)+\left({\mathrm{Max}}_{\text{iter}}\times \text{fe}\times n\right)+\left({\mathrm{Max}}_{\text{iter}}\times n\times d\right)\right)$$

Since $1\ll \left({\mathrm{Max}}_{\text{iter}}\times \text{fe}\times n\right)$, $1\ll \left({\mathrm{Max}}_{\text{iter}}\times n\times d\right)$, $\left(\text{nd}\ll {\mathrm{Max}}_{\text{iter}}\times \text{fe}\times n\right)$, and $\text{nd}\ll \left({\mathrm{Max}}_{\text{iter}}\times d\times n\right)$, Equation (12) can be simplified to Equation (12).

12 $$O\left(\text{GCRA}\right)≅O\left(\left({\mathrm{Max}}_{\text{iter}}\times \text{fe}\times n\right)+\left({\mathrm{Max}}_{\text{iter}}\times n\times d\right)\right)$$

Equation (12) means that GCRA has a linear time complexity, which implies that GCRA is a computationally efficient algorithm. This attribute of GCRA influenced the selection of comparative algorithms such as AOA, DMO, WOA, and ADMO. They all have linear time complexity. The goal here is to show the superiority of the exploration and exploitation mechanism of the proposed GCRA. The remaining comparative algorithms (LSHADEcnEpSin, LSHADE, LSHADE_SPACMA, and UMOEA) are high-performing methods in CEC competitions.

#### 3.5.2 Space complexity

The amount of memory space required by an algorithm for the effective function of the algorithm is referred to as space complexity. The complexity is contingent upon the size of the population and the dimension of the optimization problem. Hence, Equation (13) provides the space complexity of GCRA.

13 spacecomplexity≅O(n×d)

### 3.6 GCRA optimization analysis

As the evaluation of the function advances, the greater cane rats gravitate towards the universal solution, which could be food or water sources. The exploration phase is realized when they exit various shelters dispersed across their territory to scavenge and create trails. It is assumed that the alpha male retains the information about these trails, and as a result, other rats adjust their location based on this data. Furthermore, the males are aware of the breeding season and can isolate themselves from the group. The assumption is that once the group is separated during this season, the foraging activities are concentrated within areas of abundant food sources, which aids the exploitation. Therefore, the intelligent foraging trails and mating season actions are mathematically modeled to achieve the GCR algorithm design and perform the feature selection activities.

**Fig. 6:**
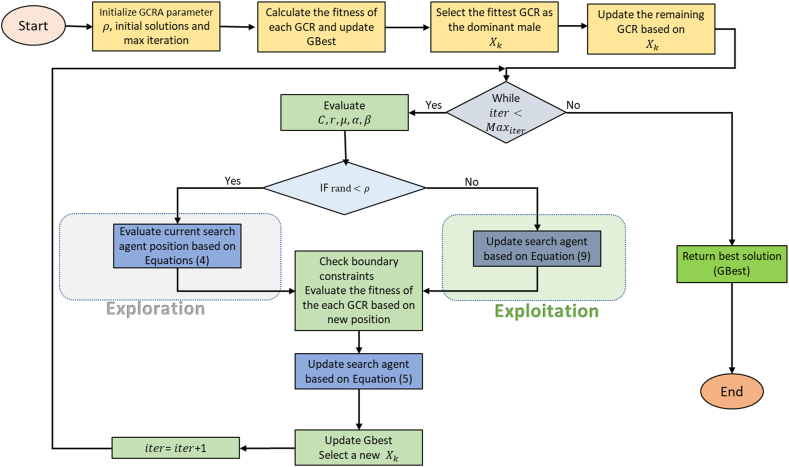
The proposed GCRA algorithmic flowchart design.

As seen from the algorithm listing in Algorithm 1, the proposed GCRA starts by randomly generating the positions of the greater rat population within the problem search boundary. Equations 4, 5, 8, 9 aim to move the GCR's initial position around the search space, looking for a global solution. The position of the GCR is evaluated using the fitness function, and the fittest GCR is the one whose position returns the minimum or maximum value of the fitness function. The optimization processes, except for the initial creation of the population, are repeated many times, determined by the maximum number of iterations. The flow chart representing the optimization process of the GCRA is shown in Fig. 6.

## 4 The experimental setup, results, and discussion

This section presents the experimental setup for the 22 classical benchmark functions, CEC 2011, and CEC 2020 problems, six (6) classical engineering problems, and the comparative results of GCRA and other state-of-the-art algorithms. The implication of the obtained results is also discussed and analyzed.

### 4.1 Experimental setup, classical benchmark functions, CEC 2020, CEC 2011, and engineering problems

The classical test functions consist of functions with different characteristics, like the shape of the landscape and the number of local and optimal solutions. They are usually classified into three categories; functions with one global solution are called unimodal functions (F1–F7). The second category, called multimodal functions (F8–F13), has multiple global solutions and high dimensions. Finally, the third category is multimodal but has a fixed low dimension (F14–F22). The CEC 2020 test suite consists of 10 functions that make finding the global optimum difficult. The detailed descriptions of both classical and CEC 2020 test functions are presented in Tables A1 and A2in the appendix, respectively.

There are 22 challenging real-world optimization problems in the CEC 2011 test suite. This set of problems was used to test the performance of GCRA. The performance of GCRA was compared with other state-of-the-art algorithms that have been used to solve this set of problems. The nature of these problems is such that they have many local optima in various regions and different shapes and dimensions. These functions and the six (6) engineering problems offer the best ways to test the ability of GCRA to avoid local optima (exploration) and find optimal or near-optimal solutions (exploitation). Details about CEC 2011 test functions can be found in Ref. [74].

The algorithms and test functions were executed using MATLAB (R2020b). The experimental setup involved a 64-bit Windows 10 OS, an Intel Core i7-7700@3.60GHz CPU, and 16G RAM. An assessment was conducted to determine the sensitivity of GCRA to variations in population size and the number of iterations. It shows that setting the number of greater cane rats to 100 and iterations to 1000 yielded the best results. As such, for each problem in the test suite, the population size and the maximum number of iterations are set to 100 and 1000, respectively. Also, the results are collated after 25 independent runs of the algorithms using four performance indicators (‘best,’ ‘worst,’ ‘mean,’ and ‘standard deviation’). The parameter settings of the algorithms used in this study are given in Table 5.

**Table 5:** Parameter settings.

Algorithm	Ref	Name of the parameter	Value of the parameter
AOA	[45]	α	5
		μ	0.05
LSHADEcnEpSin	[75]	freq_inti	0.5
		pb	0.4
		ps	0.5
CPSOGSA	[76]	<p1, <f > 2	2.05
LSHADE	[77]	p_best_rate	0.11
		memory_size	5
		arc_rate	1.4
LSHADE_SPACMA	[78]	L_Rate	0.8
		Pbest, Memory size (H), and Arc_rate	Same as LSHADE
		The threshold for SPA activated	max_nfes/2
		Probability Variable (FCP)	0.5
UMOEA	[79]	Par.MinPopSize	4
		Par.prob_ls	0.1
		PS2	4+floor(3*log(Par.n))
		PS1	Par.PopSize
WOA	[80]	a	2-t*((2)/Max_iter)
		C	2*r2
		A	2*a*r1-a
DMO	[41]	Number of babysitters	3
ADMO	[81]	Predation rate (pr)	0.5
		Birth rate (br)	0.7

**Fig. 7:**
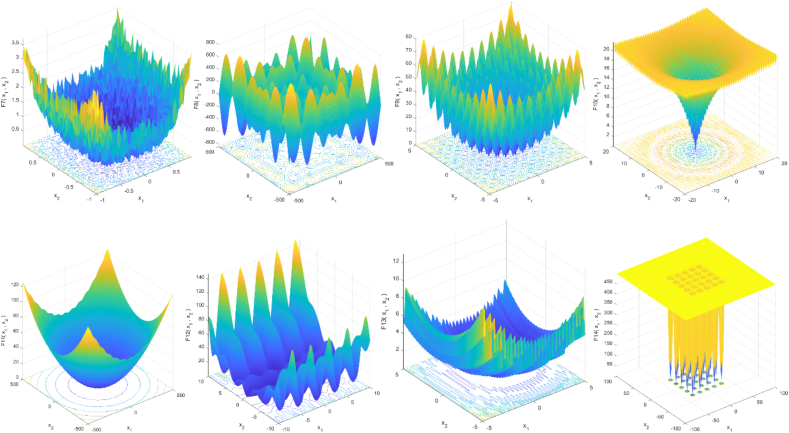
2-D representation of characteristics of some classical benchmark function.

Fig. 7 shows the graphical representation of the characteristics of some classical benchmark functions used in this study. These benchmark test functions represent some form of optimization modeled by mathematical functions. They come with parameters, dimensions, and constraints that are optimized to obtain the best possible solution. The optimal solution is mixed up with many sub-optimal solutions and then scattered around the problem search space. The problem search space or landscape consists of many hills and valleys with different complexities. The optimization algorithm's job is to find the optimal or near-optimal solution quickly. Furthermore, the robustness and efficiency of metaheuristic algorithms are measured based on their ability to achieve global search and local search leading to convergence toward the best possible solution.

The basic characteristics of the classical benchmark function are the modality, valleys, basins, dimensionality, and separability [9]. The modality defines the number of global and local solutions in the problem search space. There are two types of modality: the unimodal and multimodal functions. F7 is unimodal because it has one valley where the only global solution is located. This type of function is believed to be easy to solve, and the exploitation ability of metaheuristic algorithms is evaluated using these functions. F8–F13 are high dimensional (dynamic), and F14 is fixed multimodal functions because they have many solutions, of which only one is the global optimal, and their dimension is not fixed, it can vary. Finding the global solution is a daunting task hence, the exploratory capability of metaheuristic algorithms is tested using these functions.

### 4.2 Results and discussion

This subsection outlines the findings from the experiments conducted in this study. Initially, we delve into the results and discussions related to GCRA. This is followed by a comparative analysis of GCRA with other cutting-edge algorithms. As previously mentioned, four performance metrics (best, worst, mean, and standard deviation) are employed to encapsulate and discuss the outcomes after 25 separate executions of the algorithms. These were applied to both the 22 classical functions, 22 real-world benchmark problems, and 6 classical engineering problems. The population size and other specific algorithm metrics were kept consistent as defined in Section 4.1.

#### 4.2.1 Comparative result of GCRA for classical benchmark functions

Table 6 presents the results of GCRA and ten other state-of-the-art metaheuristic algorithms. The results show that the GCRA returned the optimal global solution for all of the 22 benchmark functions at least once, as shown by the value of the ‘Best’ indicator, and consistently found the optimal solution for 12 out of the 22 functions over 25 runs, as shown by the value of the ‘Mean, Worst, and Std’ indicators. The performance of the ADMO closely follows that of GCRA, with UMOEA, LSHADE_SPACMA, LSHADEcnEpSin, GSK, and LSHADE all showing similar competitive performance.

**Table 6:** Comparative results for classical benchmark functions.

Function	Dim	Global	Value	**GCRA**	**ADMO**	LSHADEcnEpSin	LSHADE	AOA	CPSOGSA	LSHADE_SPACMA	**WOA**	**DMO**	UMOEA	**GSK**
F1	**30**	**0**	**Best**	0	0	0	0	0	0	0	2.27E-08	0	0	0
			**Worst**	0	0	0	0	0	0	0	8.33E-06	0	0	0
			**Average**	0	0	0	0	0	0	0	1.1E-06	0	0	0
			**SD**	0	0	0	0	0	0	0	1.75E-06	0	0	0
			**Rank**	1	1	1	1	1	1	1	2	1	1	1
F2	**30**	**0**	**Best**	0	0	0	0	0	0	0	1.24E-05	0	3.29E-06	0
			**Worst**	0	0	0	0	0	0	0	0.000201	0	1.88E-05	0
			**Average**	0	0	0	0	0	0	0	7.61E-05	0	7.19E-06	0
			**SD**	0	0	0	0	0	0	0	4.81E-05	0	3.11E-06	0
			**Rank**	1	1	1	1	1	1	1	3	1	2	1
F3	**30**	**0**	**Best**	0	0	0	0	0	0	0	0.001819	0.058607	0	0
			**Worst**	0	0	0	0	0	0	0	0.020846	0.44565	0	0
			**Average**	0	0	0	0	0	0	0	0.007805	0.20423	0	0
			**SD**	0	0	0	0	0	0	0	0.004558	0.10584	0	0
			**Rank**	1	1	1	1	1	1	1	2	3	1	1
F4	**30**	**0**	**Best**	0	0	0	0	0	0	0	0.002741	5.51E-08	9.43E-06	0
			**Worst**	0	0	0	0	0	0	0	0.017679	3.26E-07	1.85E-05	1.43E-08
			**Average**	0	0	0	0	0	0	0	0.007329	1.64E-07	1.33E-05	4.75E-10
			**SD**	0	0	0	0	0	0	0	0.003554	7.93E-08	2.32E-06	2.6E-09
			**Rank**	1	1	1	1	1	1	1	4	2	3	1
F5	**30**	**0**	**Best**	0	2.62E-05	5.5019	0	4.6732	0.20837	0.25725	0.042267	0.09975	4.2179	6.1717
			**Worst**	0.000035	0.001631	8.7312	8.9902	5.8353	262.2	4.5866	15.888	6.7783	386.3	8.0797
			**Average**	0	0.000237	7.1658	7.0876	5.1486	34.932	3.4081	5.9404	1.7159	40.951	6.9003
			**SD**	0.00043	0.000306	0.91571	3.6114	0.28308	74.046	1.2584	4.0684	1.8038	85.319	0.45096
			**Rank**	1	2	9	9	5	10	4	6	3	11	8
F6	**30**	**0**	**Best**	0	0	0.002008	1.3943	0.0048	0	0	1.43E-08	0	0	0.11612
			**Worst**	0	0	0.77724	2.25	0.022207	0	0	1.95E-06	0	0	0.53614
			**Average**	0	0	0.22968	1.8804	0.012455	0	0	5.73E-07	0	0	0.27222
			**SD**	0	0	0.2326	0.18309	0.004317	0	0	5.7E-07	0	0	0.10582
			**Rank**	1	1	5	7	4	1	1	2	1	1	6
F7	**30**	**0**	**Best**	0	1E-06	8.75E-07	4.38E-07	1.14E-07	1.46E-06	1.6E-06	2.75E-07	1.37E-07	9.43E-07	3.16E-06
			**Worst**	2.21E-06	2.21E-05	0.000113	5.25E-05	7.89E-05	5.09E-05	6.91E-05	7.35E-05	6.74E-05	0.000117	0.000101
			**Average**	7.01E-06	8.01E-06	2.69E-05	1.49E-05	2.53E-05	1.93E-05	1.96E-05	2.14E-05	1.98E-05	2.63E-05	3.14E-05
			**SD**	4.05E-06	4.95E-06	2.92E-05	1.09E-05	2.33E-05	1.45E-05	1.59E-05	2.14E-05	1.74E-05	2.79E-05	2.59E-05
			**Rank**	1	2	10	3	9	5	6	8	7	11	12
F8	**30**	**−4189.8**	**Best**	−4189.8	−4189.7	−3551.7	−2392.2	−4071.4	−3558.1	−3479.2	−3972.7	−4189.8	−3498.9	−2736.2
			**Worst**	−4189.8	−4020.9	−2455.3	−1854.9	−2847.5	−2194.5	−2808	−2966	−4189.8	−2209.6	−2019.5
			**Average**	−4189.8	−4147.5	−2876.8	−2126	−3530.5	−2886	−3111.8	−3529.8	−4189.8	−2903.9	−2320.9
			**SD**	2.78E-12	57.212	279.42	120.6	272.09	348.4	194.69	285.29	2.78E-12	349.72	171.82
			**Rank**	1	2	6	3	11	7	9	10	1	8	4
F9	**30**	**0**	**Best**	0	0	0	0	0	6.9647	2.9857	2.9849	0	3.9798	0
			**Worst**	0	0	15.395	0	0	48.753	16.914	13.929	0	26.864	15.589
			**Average**	0	0	1.913	0	0	20.43	10.215	6.7989	0	14.128	0.91261
			**SD**	0	0	3.9178	0	0	10.122	3.7495	2.8752	0	7.3226	3.5087
			**Rank**	1	1	2	1	1	8	6	4	1	7	5
F10	**30**	**0**	**Best**	0	0	0	0	0	0	0	0.000104	0	6.4E-06	0
			**Worst**	0	0	3.197	0	0	0	0	0.000719	0	2.0133	0
			**Average**	0	0	0.10662	0	0	0	0	0.000301	0	0.53401	0
			**SD**	0	0	0.58369	0	0	0	0	0.000167	0	0.73626	0
			**Rank**	1	1	3	1	1	1	1	2	1	4	1
F11	**30**	**0**	**Best**	0	0	0	0	0	0.044258	0.012316	0.007398	0	0.046707	0
			**Worst**	0	0.014773	0.23598	0	0	0.74097	0.15251	0.083613	0	0.55523	0.44807
			**Average**	0	0.000492	0.01984	0	0	0.26065	0.071743	0.0472	0	0.23162	0.02325
			**SD**	0	0.002697	0.044677	0	0	0.18155	0.033792	0.020439	0	0.12381	0.083556
			**Rank**	1	2	4	1	1	9	7	6	1	8	5
F12	**30**	**0**	**Best**	0	0	0.000395	0.061063	0.000217	0	0	0	0	0	0.011676
			**Worst**	0	0	0.16786	2.4347	0.63623	1.8684	0	2.96E-07	0	1.244	0.17761
			**Average**	0	0	0.03538	0.46583	0.023788	0.30086	0	2.3E-08	0	0.062201	0.062388
			**SD**	0	0	0.038961	0.44594	0.11573	0.53298	0	6.46E-08	0	0.23671	0.037729
			**Rank**	1	1	4	8	5	0	1	2	1	6	7
F13	**30**	**0**	**Best**	0	0	0.001955	0	0.46975	0	0	0	0	0	0.15457
			**Worst**	0	1.31E-08	0.63082	0.9	0.99431	0	0	1.81E-07	0	0.010987	0.47033
			**Average**	0	8.03E-10	0.29821	0.09	0.82108	0	0	3.61E-08	0	0.000366	0.2681
			**SD**	0	3.07E-09	0.14465	0.27462	0.14167	0	0	3.79E-08	0	0.002006	0.078078
			**Rank**	1	2	7	6	9	1	1	3	1	4	8
F14	**2**	**1**	**Best**	1	0.998	0.998	0.998	2.9821	0.998	0.998	0.998	0.998	0.998	0.998
			**Worst**	0.998	0.998	4.9505	1.0057	12.671	20.153	0.998	15.504	0.998	0.998	2.9821
			**Average**	0.998	0.998	1.9895	0.99858	10.561	5.3308	0.998	4.8894	0.998	0.998	1.1965
			**SD**	0	0	1.1342	0.001822	3.1698	4.7139	0	3.798	0	2.16E-16	0.60539
			**Rank**	1	1	5	3	9	8	1	7	1	2	4
F15	**0**	**0.0003**	**Best**	0.0003	0.000307	0.000308	0.000694	0.000327	0.000355	0.000307	0.00031	0.000479	0.000317	0.000308
			**Worst**	0.000307	0.000307	0.001606	0.001512	0.10929	0.020363	0.020363	0.020363	0.00076	0.020364	0.001419
			**Average**	0.000307	0.000307	0.000522	0.000965	0.010701	0.005947	0.002087	0.002725	0.000665	0.001554	0.000957
			**SD**	1.94E-13	1.94E-13	0.000324	0.000341	0.021268	0.008843	0.004994	0.005989	7.58E-05	0.003567	0.000377
			**Rank**	1	1	2	5	11	10	7	8	3	6	4
F16	**2**	**−1.0316**	**Best**	−1.0316	−1.0316	−1.0316	−1.0316	−1.0316	−1.0316	−1.0316	−1.0316	−1.0316	−1.0316	−1.0316
			**Worst**	−1.0316	−1.0316	−1.0316	−1.0315	−1.0316	−1.0316	−1.0316	−1.0316	−1.0316	−1.0316	−1.0316
			**Average**	−1.0316	−1.0316	−1.0316	−1.0316	−1.0316	−1.0316	−1.0316	−1.0316	−1.0316	−1.0316	−1.0316
			**SD**	6.32E-16	6.39E-16	2.55E-07	3.12E-05	6.67E-08	6.32E-16	6.78E-16	1.24E-15	6.78E-16	4.76E-15	1.69E-05
			**Rank**	1	2	8	10	7	1	3	4	3	5	9
F1**7**	**2**	**3**	**Best**	3	3	3	3	3	3	3	3	3	3	3
			**Worst**	3	3	3.001	3.0001	30	3	3	30	3	3	3
			**Average**	3	3	3.0002	3	11.1	3	3	3.9	3	3	3
			**SD**	6.23E-16	8.33E-16	0.000259	2.69E-05	12.584	1.26E-15	6.23E-16	4.9295	1.97E-15	6.89E-14	3.62E-06
			**Rank**	1	2	9	8	11	3	1	10	4	5	7
F1**8**	**3**	**−3.86**	**Best**	−3.86	−3.8628	−3.8628	−3.8617	−3.8608	−3.8628	−3.8628	−3.8628	−3.8628	−3.8628	−3.862
			**Worst**	−3.8628	−3.8628	−3.8547	−3.8544	−3.8498	−3.8628	−3.8628	−3.8628	−3.8628	−3.8628	−3.8526
			**Average**	−3.8628	−3.8628	−3.8608	−3.8555	−3.854	−3.8628	−3.8628	−3.8628	−3.8628	−3.8628	−3.8559
			**SD**	2.71E-15	2.71E-15	0.003022	0.002082	0.002684	2.54E-15	2.71E-15	2.26E-15	2.71E-15	2.2E-14	0.002778
			**Rank**	1	1	6	3	4	1	1	1	1	1	5
F**19**	**6**	**−3.32**	**Best**	−3.322	−3.322	−3.3208	−3.1652	−3.2102	−3.322	−3.322	−3.322	−3.322	−3.322	−3.2088
			**Worst**	−3.322	−3.322	−2.8376	−1.9177	−3.0065	−3.2031	−3.2031	−3.2031	−3.322	−3.1987	−2.2567
			**Average**	−3.322	−3.322	−3.2445	−3.0569	−3.1086	−3.219	−3.2625	−3.2943	−3.322	−3.2224	−3.0057
			**SD**	2.28E-15	2.28E-11	0.12036	0.24041	0.055956	0.041107	0.060463	0.051146	1.43E-15	0.045329	0.1696
			**Rank**	1	1	7	10	9	2	5	4	1	3	8
F2**0**	**4**	**−10.1532**	**Best**	−10.153	−10.153	−9.9702	−8.7714	−7.5309	−10.153	−10.153	−10.153	−10.153	−10.153	−6.506
			**Worst**	−10.153	−10.153	−2.5645	−0.88199	−2.2566	−2.6305	−2.6305	−2.6305	−10.131	−2.6305	−0.49729
			**Average**	−10.153	−10.153	−5.5877	−5.5321	−4.2697	−6.4731	−7.4761	−5.4851	−10.152	−8.7275	−3.6039
			**SD**	5.41E-16	5.41E-15	2.849	1.4164	1.0722	3.3954	3.4128	3.4524	0.00406	2.6947	2.0591
			**Rank**	1	1	8	9	10	4	5	3	2	6	11
F2**1**	**4**	**−10.4028**	**Best**	−10.403	−10.403	−10.376	−8.4361	−7.2375	−10.403	−10.403	−10.403	−10.403	−10.403	−8.4693
			**Worst**	−10.403	−10.403	−2.6892	−0.90808	−1.7517	−1.8376	−2.7519	−2.7519	−10.403	−2.7519	−0.90968
			**Average**	−10.403	−10.403	−5.8629	−5.9236	−4.1539	−7.2929	−8.6769	−7.3087	−10.403	−9.6191	−4.6177
			**SD**	3.61E-15	3.63E-15	2.9202	1.4845	1.2648	3.6856	2.9752	3.4262	1.62E-08	2.0677	1.733
			**Rank**	1	1	7	9	10	6	4	5	1	3	8
F2**2**	**4**	**−10.5363**	**Best**	−10.536	−10.536	−10.393	−10.118	−7.8174	−10.536	−10.536	−10.536	−10.536	−10.536	−9.4044
			**Worst**	−10.536	−10.536	−2.4055	−5.1285	−2.1909	−2.4217	−2.4217	−1.8595	−10.536	−5.1285	−0.94553
			**Average**	−10.536	−10.536	−5.7166	−6.371	−4.4501	−7.0219	−8.8234	−7.1483	−10.536	−9.9988	−5.1252
			**SD**	2.6E-15	3.76E-14	2.5849	1.3998	1.7161	3.8783	3.2147	3.7315	2.6E-15	1.6405	1.9278
			**Rank**	1	1	7	8	10	6	4	5	1	2	9
Mean rank				4.5	4.48	6.34	6.95	7.18	5.68	5.48	6.43	5.09	6.39	7.48
Rank				1	2	8	6	9	6	4	9	3	7	10

Specifically, the GCRA showed consistent performance for the unimodal problems (F1–F7). These functions are excellent in testing the exploitation ability of algorithms. The GCRA successfully found global solutions for these functions, which confirms the exploitation ability of GCRA. Also, the GCRA performed exceptionally for the multimodal functions (F8–F22), which are good in testing the exploration ability of the metaheuristic algorithm. Occasioned by the number of these functions, the GCRA successfully found global solutions, it can be concluded that the exploratory ability of GCRA is excellent.

Utilizing Friedman's test for ranking, it was observed that the proposed GCRA secured the top position with the least mean value, closely trailed by ADMO and UMOEA. The Wilcoxon's signed rank test was employed to juxtapose the outcomes of all considered algorithms, as depicted in Table 7. The superior performance of GCRA is further substantiated as it notably surpasses DMO, AOA, WOA, and CPSOGSA across all evaluated functions, as evidenced by the high value of the positive ranks returned. Moreover, the number of ties yielded by the comparisons of GCRA with ADMO, UMOEA, LSHADE_SPACMA, LSHADEcnEpSin, GSK, and LSHADE suggests the competitive nature of their results. The findings suggest that GCRA notably excelled over half of the algorithms, while its superiority over the remaining five was less pronounced. Consequently, it can be inferred that GCRA serves as an efficient instrument for tackling this array of optimization issues, striking a balance between exploration and exploitation.

**Table 7:** Comparative results of Wilcoxon's test for classical benchmark functions.

Algorithm		N	Mean Rank	Sum of Ranks	P-value	Asymp. Sig. (2-tailed)
ADMO - GCRA	Negative Ranks	2	4.50	9.00	−0.314b	0.753
	Positive Ranks	4	3.00	12.00		
	Ties	16				
	Total	22				
LSHADEcnEpSin - GCRA	Negative Ranks	2	7.00	14.00	−2.201b	0.028
	Positive Ranks	11	7.00	77.00		
	Ties	9				
	Total	22				
LSHADE - GCRA	Negative Ranks	2	3.50	7.00	−2.312b	0.021
	Positive Ranks	9	6.56	59.00		
	Ties	11				
	Total	22				
DMO - GCRA	Negative Ranks	1	4.00	4.00	−2.900b	0.004
	Positive Ranks	12	7.25	87.00		
	Ties	9				
	Total	22				
LSHADE_SPACMA - GCRA	Negative Ranks	2	3.50	7.00	−1.540b	0.123
	Positive Ranks	6	4.83	29.00		
	Ties	14				
	Total	22				
UMOEA - GCRA	Negative Ranks	2	3.50	7.00	−1.540b	0.123
	Positive Ranks	6	4.83	29.00		
	Ties	14				
	Total	22				
WOA - GCRA	Negative Ranks	2	9.00	18.00	−2.166b	0.03
	Positive Ranks	12	7.25	87.00		
	Ties	8				
	Total	22				
AOA - GCRA	Negative Ranks	2	4.50	9.00	−0.845b	0.398
	Positive Ranks	5	3.80	19.00		
	Ties	15				
	Total	22				
CPSOGSA - GCRA	Negative Ranks	2	6.50	13.00	−1.778b	0.075
	Positive Ranks	9	5.89	53.00		
	Ties	11				
	Total	22				
GSK - GCRA	Negative Ranks	2	3.50	7.00	−2.691b	0.007
	Positive Ranks	11	7.64	84.00		
	Ties	9				
	Total	22				

b. Based on negative ranks.

#### 4.2.2 Results for GCRA for CEC 2020 functions

This subsection showcases the GCRA's performance in resolving the CEC 2020 complex functions. A comparison of GCRA's performance with ten other algorithms is made, with the outcomes detailed in Table 8. Out of the ten functions in the test suite, GCRA identified the optimal solution for four. It notably surpassed the rival algorithms used in this research, securing the top rank. The AOA and WOA algorithms demonstrated the poorest performance for this set of problems. Judging by the average fitness value and standard deviation, GCRA recorded the lowest figures among all algorithms, underscoring its stability in addressing these issues.

**Table 8:** Comparative results for CEC 2020 test functions.

Function	Global	Value	GCRA	AOA	LSHADEcnEpSin	LSHADE	ADMO	CPSOGSA	LSHADE_SPACMA	WOA	DMO	UMOEA	GSK
F1	**100**	**Best**	100	1.02E+07	104.2	1.81E+09	100.69	100.47	100.96	142.99	107.53	2.44E+08	140.8
		**Worst**	101.39	4.12E+09	11441	1.29E+10	12694	5621.7	5494.9	7359.8	12243	1.29E+09	5.07E+08
		**Average**	100.4	7.19E+08	2853.6	6.54E+09	4722.1	1692.5	876.9	2795	3236	7.06E+08	1.89E+07
		**SD**	0.3515	1.20E+09	2966.3	2.57E+09	4289.2	1682.6	1302.9	2097.7	2872.5	2.98E+08	9.23E+07
		**Rank**	1	9	5	11	3	2	4	7	6	10	8
F2	**1100**	**Best**	1130.2	1451.2	1126.9	1359.4	1594.2	1118.5	1115.2	1160.7	1138.5	1552.1	1112.2
		**Worst**	1375.1	2666.7	2567.9	2707.7	2832	1823.1	2263.4	1724.3	2333.4	2600.6	2062.1
		**Average**	1241.4	2194.6	1709.7	2021.1	2034.2	1453.2	1590.8	1552.1	1766.4	2269.5	1513.2
		**SD**	63.833	288.25	305.26	288.74	298.97	171.22	281.95	131.95	262.51	275.68	208.43
		**Rank**	1	10	6	8	9	2	5	4	7	11	3
F3	**700**	**Best**	714.01	747.21	720.96	769.12	720.77	711.97	714.7	717.27	716.57	753.67	713.28
		**Worst**	724.97	835.07	799.36	834.54	883.67	729.72	730.06	726.09	772.57	799.94	754.41
		**Average**	719.69	778.54	745.26	797.74	761.4	720.38	720.67	722.33	732.7	774.21	733.63
		**SD**	2.963	19.441	22.3	15.613	31.878	3.7772	4.1885	2.1465	12.868	9.7638	11.536
		**Rank**	1	10	7	11	8	2	3	4	5	9	6
F4	**1900**	**Best**	1900.2	1903.1	1900.8	2571.8	1900.1	1900.5	1900.5	1901	1900.4	1909.1	1900.7
		**Worst**	1901.8	27556	1916	2.00E+05	1906.5	1901.5	1905.1	1901.9	1903.4	2048.7	1903.5
		**Average**	1901.2	3264.3	1905.3	52046	1901.8	1901	1901.5	1901.6	1901.5	1926.5	1902
		**SD**	0.3165	5087.8	2.9321	57296	1.2935	0.28017	0.93587	0.22955	0.73147	27.946	0.80342
		**Rank**	1	8	6	9	4	1	2	3	3	7	5
F5	**1700**	**Best**	1710.1	3674.5	1868.4	3679.4	2268.2	1957.2	2682.9	3655.3	3015	6282	2695.1
		**Worst**	1797.9	5.71E+05	5.67E+05	3.10E+05	1.22E+05	18436	2.18E+05	79843	20479	84903	3.47E+05
		**Average**	1751.3	69217	84962	1.55E+05	19498	6326.5	39375	24258	6712.5	22104	17781
		**SD**	16.644	1.63E+05	1.69E+05	70051	30567	4401.8	57716	19590	3874.4	18350	62170
		**Rank**	1	9	10	11	5	2	8	7	3	6	4
F6	**1600**	**Best**	1600.2	1600.9	1600.5	1601.1	1600.5	1600.5	1600.5	1600.5	1600.5	1600.8	1600.8
		**Worst**	1600.5	1659.5	1619.5	1628.8	1727.1	1659.5	1618	1600.8	1601.6	1602.1	1617.5
		**Average**	1600.5	1606.2	1603.7	1615.1	1626.1	1617.2	1603.2	1600.6	1601	1601.5	1602.9
		**SD**	0.001129	12.062	5.708	8.062	34.97	20.581	5.8731	0.073957	0.29051	0.28056	5.1751
		**Rank**	1	8	7	9	11	10	6	2	3	4	5
F7	**2100**	**Best**	2101.1	4674	2102.1	3334.3	2891.9	2101.8	2121.4	2208.8	2438.6	4020	2450.9
		**Worst**	2122.5	27880	26801	10767	23780	5640.2	24664	5785	21675	19690	19648
		**Average**	2108	11226	9730.3	6361.5	9699.7	2741.6	9666.2	3097.4	7310	9433.6	9618
		**SD**	4.5969	6225.9	7716.8	2263.6	7186.9	746.11	7064.8	1025.2	5184.9	4470.4	5057.1
		**Rank**	1	11	10	4	9	2	8	3	7	5	6
F8	**2200**	**Best**	2200	2238.5	2238.3	2456.4	2301.1	2301.1	2222.3	2236.9	2222.9	2332.3	2300.9
		**Worst**	2304.2	2544.7	2321.4	3388.6	4219.3	2305.1	2304.2	2301.5	2305.7	2424.8	2327.9
		**Average**	2278.3	2362.6	2302.8	2770.9	2428.8	2302.4	2299.4	2295.9	2295.9	2368.9	2307.5
		**SD**	43.878	71.069	21.088	237.13	424.81	1.0982	14.607	15.238	23.22	22.146	6.9467
		**Rank**	1	7	5	9	10	4	3	2	2	8	6
F9	**2400**	**Best**	2500	2521.9	2500	2679.8	2500	2500	2500	2599	2722.3	2552.3	2501.3
		**Worst**	2739.7	2830.3	2830.6	2975.7	2804.9	2753.9	2773.4	2753.2	2784.5	2794.4	2777.8
		**Average**	2559.5	2733.4	2781.8	2823.7	2743.4	2717.7	2722.1	2732	2745.1	2772.6	2736.7
		**SD**	83.741	97.457	56.986	66.405	83.431	74.071	75.875	36.434	10.568	42.3	47.034
		**Rank**	1	5	10	11	7	2	3	4	8	9	6
F10	**2500**	**Best**	2602.3	2901	2600.1	2908.4	2897.7	2898.1	2897.7	2900	2897.8	2926	2902.6
		**Worst**	2897.8	3055.1	3024.3	3433.7	3024.2	2946	2948.9	2946.2	2951.3	3041.5	2949.5
		**Average**	2887.9	2955.5	2938.1	3121.4	2931.6	2924.7	2930.5	2918.1	2924.2	2959.9	2936.8
		**SD**	53.863	38.61	72.682	113.74	30.553	23.104	23.126	16.173	24.521	23.205	14.062
		**Rank**	1	9	8	11	7	4	5	2	3	10	6
Mean rank			1.10	9.00	7.70	9.80	7.80	3.30	4.75	3.75	4.60	8.20	6.00
Rank			1	10	7	11	8	2	5	3	4	9	6

#### 4.2.3 Results for GCRA for CEC 2011 problems

The GCRA was employed to solve the 22 real-world problems outlined in the CEC 2011 test suite, with the findings detailed in Table 9. Unlike the benchmark problems defined in other CEC competitions, this problem set does not provide an optimal value or solution. The table reveals that for F3, F4, F8, and F10, GCRA consistently found the same value across 25 independent algorithm runs. This consistency suggests that the discovered value could be the sought-after global solution for these problems. It's also noted that the 'Best' indicator's value is consistently lower than the 'Worst' and 'Mean' indicators' values. This pattern indicates that GCRA could discover a variety of solutions, effectively exploring the search space and avoiding local optima. The small 'Std' values signify GCRA's stability around the results. This stability and performance affirm GCRA's effectiveness, robustness, and stability in optimizing these problems. The next step involves comparing GCRA's performance with other cutting-edge algorithms to further assess its superiority and robustness.

**Table 9:** Results of GCRA for CEC 2011 problems.

Function	Best	Worst	Mean	Std
F1	0.00E+00	8.25E-02	4.06E-03	1.51E-02
F2	−2.83E+01	−2.71E+01	−2.84E+01	2.01E-01
F3	1.15E-05	1.15E-05	1.15E-05	6.08E-11
F4	0.00E+00	0.00E+00	0.00E+00	0.00E+00
F5	−3.78E+01	−3.59E+01	−3.68E+01	7.33E-01
F6	−2.99E+01	−1.82E+01	−2.70E+01	4.04E+00
F7	8.70E-01	1.30E+00	1.14E+00	1.02E-01
F8	2.19E+02	2.19E+02	2.19E+02	0.00E+00
F9	3.00E+05	3.01E+05	3.00E+05	1.83E+00
F10	−6.80E+00	−6.80E+00	−6.80E+00	0.00E+00
F11	5.09E+04	5.30E+04	5.22E+04	6.27E+02
F12	1.06E+06	1.07E+06	1.06E+06	1.41E+03
F13	1.54E+04	1.54E+04	1.54E+04	2.61E-06
F14	1.79E+04	1.83E+04	1.80E+04	3.09E+01
F15	3.27E+04	3.27E+04	3.27E+04	5.87E-01
F16	1.23E+05	1.27E+05	1.24E+05	9.07E+02
F17	1.85E+06	1.90E+06	1.89E+06	1.27E+04
F18	9.35E+05	9.44E+05	9.41E+05	3.00E+03
F19	9.42E+05	9.59E+05	9.47E+05	4.58E+03
F20	9.36E+05	9.49E+05	9.40E+05	3.29E+03
F21	1.26E+01	1.79E+01	1.51E+01	1.27E+00
F22	1.19E+01	1.31E+01	1.27E+01	1.49E+00

**Fig. 8:**
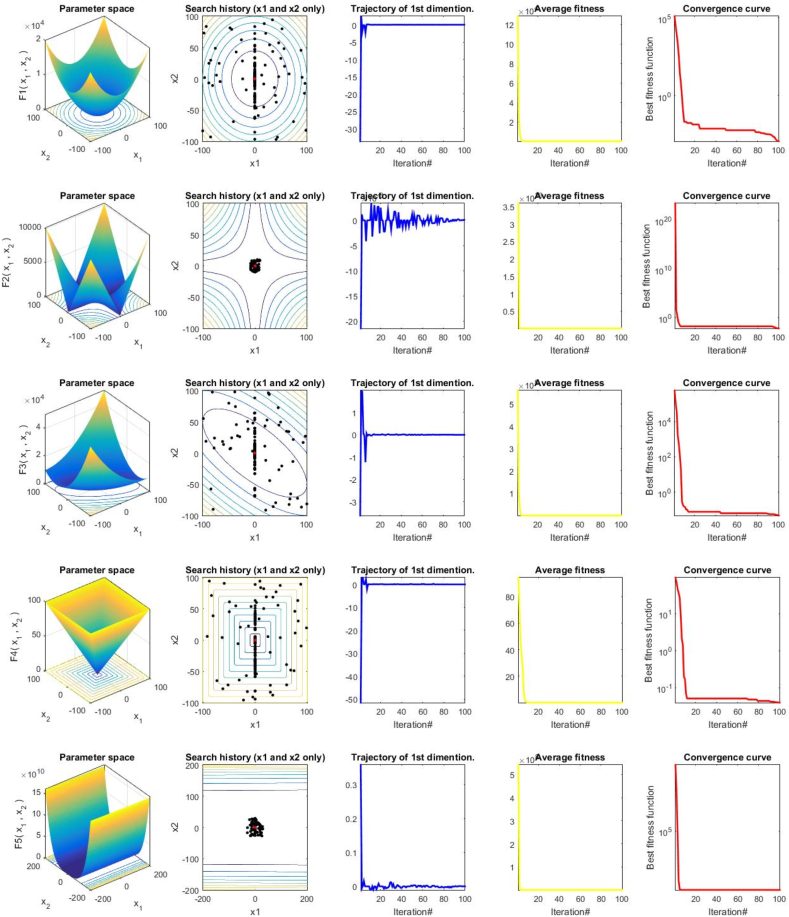

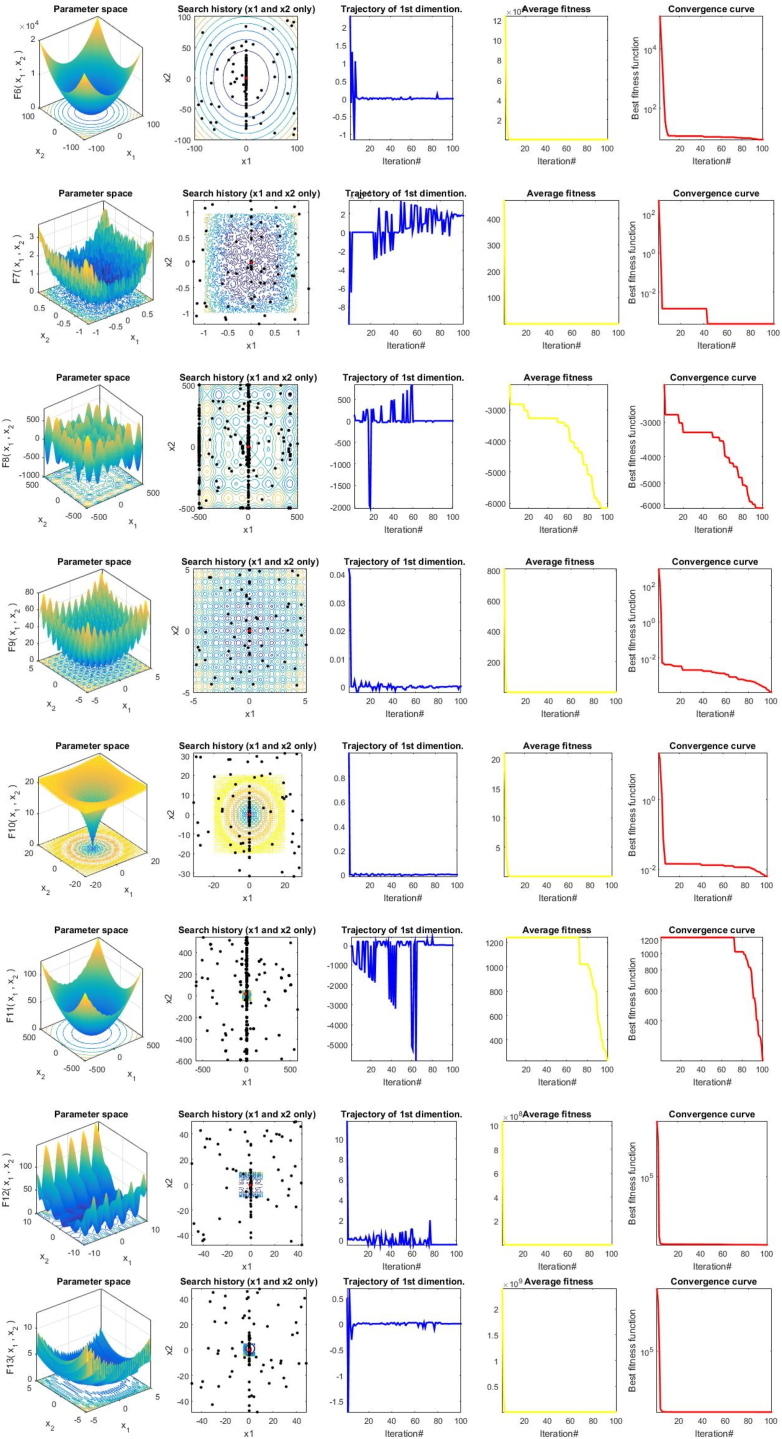
Qualitative results for some of the studied benchmark problems from F1–F13.

Moreover, to prove the convergence behavior of the proposed GCRA metaheuristic optimizer, several evaluation metrics were employed in two-dimensional space to clearly illustrate the spectacular unique characteristics of the GCRA performance evolution. These 2D evaluations that are depicted in Fig. 8 include search history, search trajectory, average fitness, and convergence curve behavior. The graphs equally demonstrate the quantitative results of GCRA exploration, exploitation, and self-improvement of the initial population. Also, note that the trajectory and convergence curves shown in the third and fifth columns are often used to evaluate the convergence performance and capability of the GCRA optimization method to handle complex or robust optimization problems.

The convergence curves of all the functions depicted here, which have one global optimum, are very smooth and drop rapidly compared to those with rough surfaces such as F7, F8, and F9. Therefore, because of this smoothness, the convergence can reach a stable value only after the first few iterations. This proves the effectiveness of the movement strategy for exploitation in the GCRA [82]. However, in the case of the remaining functions, whose curves are not smooth, the skill of exploration is more biased than the skill of exploitation. However, this does not imply that the convergence curves cannot, in all cases, accurately approximate the global optimum value in the final iteration stage of the algorithm iterative process.

#### 4.2.4 Comparative results for CEC 2011 problems

Table 10 compares the results of GCRA with some of the state-of-the-art algorithms. In this table, two performance indices (‘Mean’ and ‘Std’) were used to compare the results. The choice of algorithms is motivated basically by their performance in CEC competitions, solving other optimization problems, and the availability of source code online. For instance, the LSHADE, LSHADEcnEpSin, LSHADE_SPACMA, and UMOEA were high performers in different CEC competitions. Selected algorithms from other classifications or categories of metaheuristic algorithms were considered for comparison. The WOA, DMO, and ADMO are candidate representatives of the swarm-based algorithm, the gaining-sharing knowledge (GSK) algorithm [43] represents the human activity-based algorithms, and AOA and CPSOGSA represent physical-based algorithms.

**Table 10:** Comparative results for CEC 2011 problems.

Algorithms											LSHADE_SPACMA				WOA						GSK	
Function	Mean	Std	Mean	Std	Mean	Std	Mean	Std	Mean	Std	Mean	Std	Mean	Std	Mean	Std	Mean	Std	Mean	Std	3.28E+00	5.21E+00
F1	4.06E-03	1.51E-02	4.56E-03	1.64E-02	1.10E+01	1.61E+00	1.61E+00	3.34E+00	1.76E+01	5.78E+00	1.12E+01	6.61E+00	6.22E-01	2.41E+00	1.93E+01	5.65E+00	2.50E+01	4.44E+00	1.81E+01	5.25E+00	−1.13E+01	1.03E+00
F2	−2.84E+01	2.01E-01	−2.84E+01	2.17E-01	−1.46E+01	2.33E+00	−2.60E+01	1.72E+00	−2.48E+01	1.84E+00	−2.83E+01	3.94E-01	−2.83E+01	3.31E-01	−2.51E+01	2.17E+00	−8.03E+00	1.37E+00	−3.82E+00	3.14E+00	1.15E-05	9.12E-13
F3	1.15E-05	6.08E-11	1.15E-05	6.98E-11	1.15E-05	8.90E-12	1.15E-05	8.75E-13	2.03E-01	1.23E-02	1.15E-05	6.78E-09	1.15E-05	9.07E-10	1.22E-01	2.45E-01	1.23E+00	1.09E+00	2.01E-02	1.09E-03	0.00E+00	0.00E+00
F4	0.00E+00	0.00E+00	0.00E+00	0.00E+00	0.00E+00	0.00E+00	0.00E+00	0.00E+00	0.00E+00	0.00E+00	0.00E+00	0.00E+00	0.00E+00	0.00E+00	0.00E+00	0.00E+00	0.00E+00	0.00E+00	0.00E+00	0.00E+00	−2.06E+01	1.21E+00
F5	−3.68E+01	7.33E-01	−3.58E+01	7.43E-01	1.00E+30	2.89E+14	−3.24E+01	1.22E+00	−2.39E+01	2.18E+00	−3.59E+01	6.69E-01	−3.57E+01	1.05E+00	−2.81E+01	3.46E+00	−2.10E+01	1.07E+00	−2.02E+01	7.38E+00	−6.94E+00	2.48E+00
F6	−2.70E+01	4.04E+00	−2.60E+01	4.44E+00	1.00E+30	2.89E+14	−2.63E+01	1.46E+00	−1.71E+01	2.36E+00	−2.91E+01	2.07E-01	−2.91E+01	1.64E-01	−1.91E+01	2.94E+00	−1.40E+01	1.22E+00	−1.24E+01	6.44E+00	1.78E+00	1.08E-01
F7	1.14E+00	1.02E-01	1.15E+00	1.08E-01	1.06E+00	7.92E-02	1.13E+00	1.54E-01	1.61E+00	1.73E-01	1.27E+00	8.82E-02	5.59E-01	9.95E-02	1.72E+00	1.97E-01	1.84E+00	7.80E-02	8.35E-01	1.81E-01	2.20E+02	0.00E+00
F8	2.19E+02	0.00E+00	2.20E+02	0.00E+00	2.20E+02	0.00E+00	2.20E+02	0.00E+00	2.22E+02	6.93E+00	2.51E+02	1.49E+01	2.20E+02	0.00E+00	2.56E+02	3.40E+01	2.85E+02	2.33E+01	3.02E+02	6.05E+01	2.11E+03	5.02E+02
F9	3.00E+05	1.83E+00	3.01E+05	1.88E+00	1.69E+05	1.01E+04	4.72E+05	8.86E+04	1.66E+06	7.50E+04	3.01E+05	3.02E+02	1.05E+06	1.66E+05	1.03E+06	4.73E+04	4.51E+06	2.26E+05	3.05E+05	9.21E+02	−2.16E+01	1.19E-01
F10	−6.80E+00	0.00E+00	−6.80E+00	0.00E+00	−2.17E+01	1.20E-01	−2.11E+01	2.01E-01	−1.12E+01	7.86E-01	−9.02E+00	5.24E+00	−1.11E+01	9.14E-01	−1.07E+01	7.80E-01	−1.09E+01	1.62E+00	−1.49E+01	2.30E+00	5.24E+04	6.88E+02
F11	5.22E+04	6.27E+02	5.24E+04	6.47E+02	5.21E+04	5.48E+02	9.23E+05	2.55E+05	3.97E+05	1.19E+05	8.55E+06	2.49E+05	3.19E+08	2.61E+07	1.27E+06	1.07E+05	6.42E+06	6.83E+04	9.84E+05	3.46E+05	1.07E+06	1.73E+03
F12	1.06E+06	1.41E+03	1.07E+06	1.44E+03	1.08E+06	9.44E+03	3.32E+06	6.23E+05	4.68E+06	4.60E+05	1.00E+30	1.48E+14	5.20E+06	3.71E+05	1.47E+07	7.88E+05	1.24E+06	1.07E+05	1.10E+06	3.15E+03	1.54E+04	2.44E+00
F13	1.54E+04	2.61E-06	1.54E+04	2.63E-06	1.54E+04	1.39E+00	1.54E+04	1.97E-01	1.55E+04	2.79E+01	1.00E+30	1.48E+14	1.55E+04	6.99E+00	1.56E+04	5.46E+01	1.56E+04	1.15E+02	1.55E+04	2.13E+01	1.84E+04	1.22E+02
F14	1.80E+04	3.09E+01	1.81E+04	3.99E+01	1.81E+04	3.37E+01	1.85E+04	3.71E+01	1.92E+04	2.58E+02	7.00E+29	4.83E+29	1.81E+04	4.52E+01	1.93E+04	2.12E+02	1.90E+04	1.52E+02	1.93E+04	1.86E+02	3.28E+04	1.55E+01
F15	3.27E+04	5.87E-01	3.27E+04	5.91E-01	3.28E+04	1.43E+01	3.28E+04	3.16E+01	3.32E+04	1.28E+02	1.00E+30	1.48E+14	5.13E+06	3.44E+06	4.06E+04	2.34E+04	3.37E+04	1.37E+03	3.31E+04	1.35E+02	1.35E+05	2.22E+03
F16	1.24E+05	9.07E+02	1.27E+05	9.39E+02	1.28E+05	8.99E+02	1.30E+05	7.07E+02	1.46E+05	7.97E+03	1.00E+30	1.48E+14	6.12E+07	1.51E+07	1.47E+05	6.99E+03	1.48E+05	3.58E+03	1.48E+05	3.82E+03	2.09E+06	1.20E+05
F17	1.89E+06	1.27E+04	1.90E+06	1.57E+04	1.90E+06	1.02E+04	1.92E+06	1.64E+04	1.56E+09	1.47E+09	1.00E+30	1.48E+14	1.86E+10	4.55E+09	1.01E+10	3.64E+09	1.40E+10	1.17E+09	2.18E+06	2.73E+05	1.27E+06	7.56E+04
F18	9.41E+05	3.00E+03	9.41E+05	3.01E+03	9.43E+05	3.27E+03	9.47E+05	3.68E+03	3.08E+06	9.94E+05	9.40E+05	1.15E+03	1.55E+08	2.01E+07	4.89E+06	5.36E+06	5.91E+07	8.67E+06	9.52E+05	5.86E+03	2.00E+06	1.36E+05
F19	9.47E+05	4.58E+03	9.48E+05	4.98E+03	9.45E+05	2.37E+03	1.22E+06	7.50E+04	4.24E+06	2.17E+06	9.44E+05	1.80E+03	1.50E+08	1.66E+07	6.57E+06	5.33E+06	5.94E+07	1.27E+07	1.39E+06	2.08E+05	1.29E+06	9.20E+04
F20	9.40E+05	3.29E+03	9.40E+05	3.27E+03	9.40E+05	2.31E+03	9.52E+05	9.28E+03	3.66E+06	1.75E+06	9.40E+05	2.05E+03	1.51E+08	1.38E+07	5.28E+06	3.00E+06	5.62E+07	1.01E+07	1.09E+06	3.50E+05	1.70E+01	3.11E+00
F21	1.51E+01	1.27E+00	1.51E+01	1.25E+00	1.51E+01	5.97E-01	1.43E+01	2.56E+00	1.00E+30	1.48E+14	4.06E+01	4.63E+00	4.55E+01	3.54E+00	2.58E+01	8.47E+00	1.81E+01	1.20E+00	2.37E+01	7.12E+00	1.29E+01	2.93E+00
F22	1.27E+01	1.49E+00	1.27E+01	1.48E+00	1.43E+01	2.13E+00	1.66E+01	1.15E+00	3.89E+01	8.73E+00	4.21E+01	3.71E+00	3.49E+01	3.17E+00	2.22E+01	3.16E+00	2.49E+01	2.43E+00	3.80E+01	6.50E+00	3.28E+00	5.21E+00

The presented results show that GCRA competed with the high performers over all the problems. Specifically, GCRA returned the same values for all the indicators with LSHADE, LSHADEcnEpSin, LSHADE_SPACMA, GSK, and UMOEA for F3, F4, F8, and F10. This scenario confirms that the value obtained is the optimal solution for the four problems. The results obtained by GCRA were very competitive for the rest of the function. However, below-par performance was recorded by the AOA, DMO, and WOA for this set of problems, competing only for F4.

**Fig. 9:**
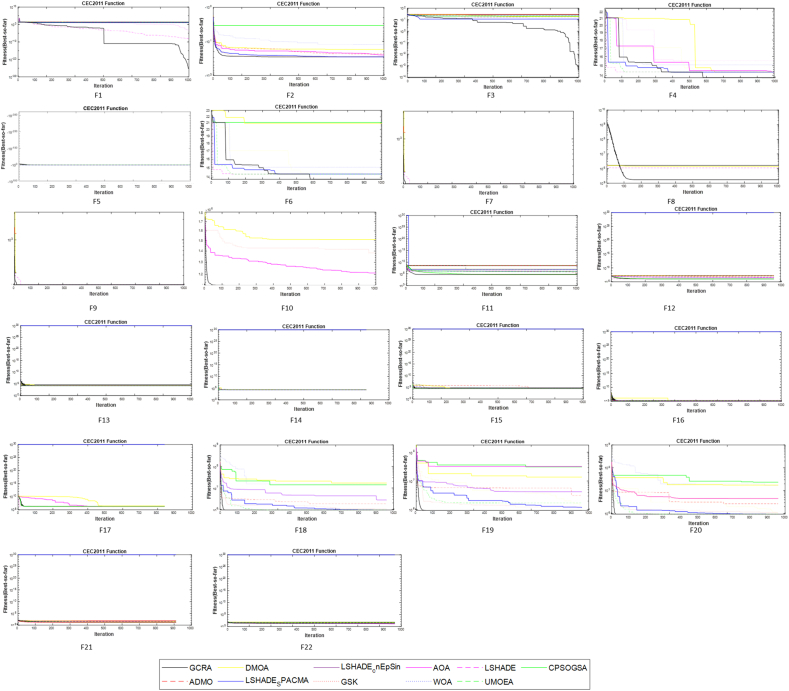
Convergence analysis.

The convergence behavior of the algorithms as they solve this set of problems is shown in Fig. 9. The GCRA and the high performers showed fast and steady convergence during the first few iterations. It remained steady around the best solution afterward. This scenario implies effective exploration early in the iteration and exploitation later during the iteration. In all cases, maintain a balance between exploration and exploitation. Specifically, the GCRA shows that quality solutions are found with a few iterations. However, the exploration and exploitation phases are continuous throughout the optimization process.

#### 4.2.5 Results for classic engineering optimization problems

Optimization algorithms’ role is crucial in the engineering domain, as they help balance the operational characteristics of objects to reduce work costs. In this context, the outcomes of optimization for 6 classical examples are presented. The comparison of the GCRA method with other meta-heuristic strategies and some standard results obtained from analytical methods (if available). This comparison is intended to underscore the efficiency and effectiveness of the GCRA method in tackling intricate engineering problems. The findings could offer valuable perspectives for the future use of optimization algorithms in the field of engineering.

##### 4.2.5.1 Three-bar truss problem

**Fig. 10:**
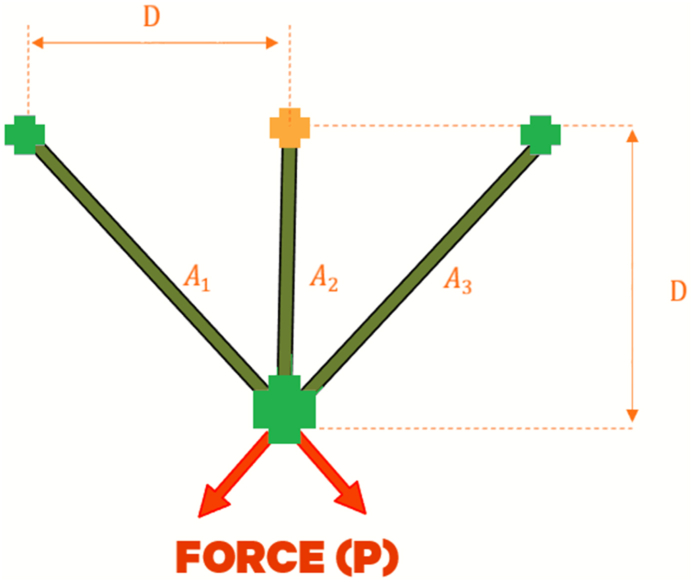
The 3-BTD.

The objective of optimizing the 3-bar truss engineering problem, illustrated in Fig. 10, is to reduce the weight of the 3-bar structure while it supports a total load P exerted vertically downward. The static volume of the loaded 3-bar truss is constrained by the stress (σ) of each bar. The 3-Bar Truss Design (3-BTD) has two design variables, namely the cross-sectional areas, $A_{1}\left(=x_{1}\right)\text{and}\;A_{2}\left(=x_{2}\right)$, as indicated in Fig. 10. Equation (14) presents the mathematical model for the 3-BTD [83].

14 $$\min\;f\left(X\right)=\left(2\sqrt{2}x_{1}+x_{2}\right)\times l$$

Subject to

$$g_{1}\left(X\right)=\frac{\sqrt{2}x_{1}+x_{2}}{\sqrt{2}x_{1}^{2}+2x_{1}x_{2}}P-\sigma \leq 0,g_{2}\left(X\right)=\frac{x_{2}}{\sqrt{2}x_{1}^{2}+2x_{1}x_{2}}P-\sigma \leq 0,g_{3}\left(X\right)=\frac{1}{\sqrt{2}x_{2}+x_{1}}P-\sigma \leq 0$$

$l=100\text{cm}.\;P=2\text{kN}/{\text{cm}}^{3}\text{,}$ $\sigma =2\text{kN}/{\text{cm}}^{3}\text{,}$ range: $0\leq x_{1},x_{2}\leq 1$ [83]

Table 11 showcases the outcomes of the optimization process performed on this problem using prospective metaheuristic algorithms.

**Table 11:** Results for 3-bar truss problem.

Algorithms	GCRA2	LSHADEcnEpSin	GOA	LSHADE	HHO	LSHADE_SPACMA	WOA	CPSOGSA	RFO [84]
x1	0.209515	0.21949849	0.214256	0.234256	0.239498	0.244256176	0.48867	0.226256	0.75356
x2	0.178373	0.178372818	0.192457	0.198424	0.198373	0.39245187	0.507569	0.193452	0.55373
Best	106.93	−47727	106.93	1.00E+30	106.93	1.00E+30	106.93	107.56	268.512
Worst	106.96	−38638	106.93	1.00E+30	106.93	1.00E+30	106.93	123.89	NA
Average	106.94	−42419	106.93	1.00E+30	106.93	1.00E+30	106.93	112.07	NA
Stan.Div	0.006604	3168.9	2.22E-10	2.89E+14	2.11E-14	2.89E+14	5.22E-07	4.0699	NA

##### 4.2.5.2 Gear train problem

Optimizing the gear train design problem is indeed an interesting one in the field of mechanical engineering. It's an unconstrained discrete design optimization problem, where the goal is to minimize the ratio of the output to the input shaft's angular velocity. The design variables in this problem are the number of teeth on each gear, represented as follows: $n_{A}\left(=x_{1}\right),n_{B}\left(=x_{2}\right),n_{C}\left({=x}_{3}\right),n_{D}\left(=x_{4}\right)$

**Fig. 11:**
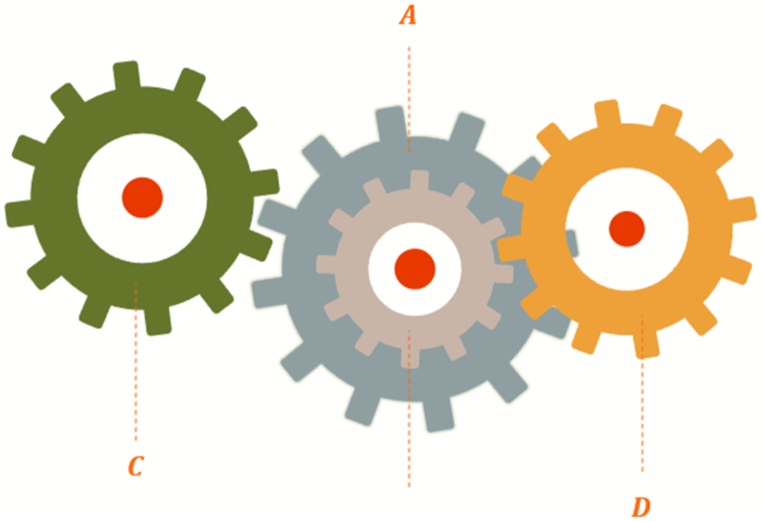
The GTD

The mathematical model for the GTD problem would be represented in Equation (15) [85]. The representation of this problem is shown in Fig. 11.

15 $$\min\;f\left(X\right)={\left(\frac{1}{6.931}-\frac{x_{3}x_{2}}{x_{1}x_{4}}\right)}^{2}$$

where.

$x_{1},x_{2},x_{3},x_{4}\in \left\{12,13,14,\ldots ,60\right\}$

The obtained results from different candidate metaheuristic algorithms are presented in Table 12.

**Table 12:** Results for gear train problem.

Algorithms	GCRA2	LSHADEcnEpSin	GOA	LSHADE	HHO	LSHADE_SPACMA	WOA	CPSOGSA	RFO [84]	PBO [82]
x1	55	48	49	34	53	43	49	49	52.1581	50.04797
x2	16	19	19	13	26	16	19	19	23.01251	23.32433
x3	16	19	16	20	15	34	19	34	16.1401	14.82582
x4	43	52	43	53	51	43	49	49	47.2105	47.88919
Best	2.70E-12	5.57E-15	2.70E-12	0.73226	2.70E-12	0.73226	2.70E-12	1.17E-10	0.08709	1.37 x 10-15
Worst	2.31E-11	4.33E-10	0.003105	0.73226	3.64E-09	0.73226	3.30E-09	2.73E-08	NA	NA
Average	1.29E-11	5.93E-11	0.000155	0.73226	7.64E-10	0.73226	1.27E-09	9.43E-09	NA	NA
Stan.Div	1.05E-11	1.17E-10	0.000694	0	9.62E-10	0	1.03E-09	1.10E-08	NA	NA

##### 4.2.5.3 Welded beam design problem

**Fig. 12:**
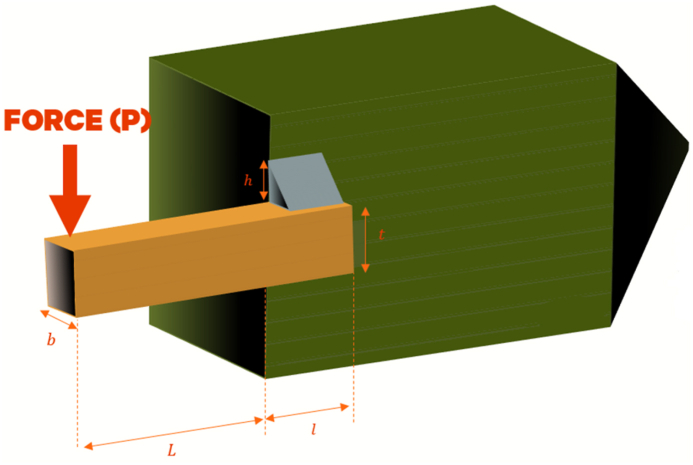
The WBD

The Welded Beam Design (WBD) problem, a well-known optimization issue, was employed to assess the effectiveness of GCRA and various other optimization algorithms. As shown in Fig. 12, the WBD problem involves designing a welded beam under several constraints, specifically shear stress (τ), beam blending stress (θ), bar buckling load (P_c_), and beam end deflection (δ). to subjected various constraints namely, shear (τ) and beam blending (θ) stress, bar buckling load ($P_{c}$) beam end deflection (δ). The objective is to reduce the production cost of the design, as illustrated in equation (16). The design variables are $h=x_{1},l=x_{2},t=x_{3},b=x_{4}$ where l is the length, h is the height, t is the thickness, and b is the weld thickness of the bar [86]

16 $$\min\;f\left(\;X\right)=x_{1}^{2}x_{2}1.10471+0.04811x_{3}x_{4}\left(14.0+x_{2}\right)$$

$$s_{1}\left(\;X\right)=\tau \left(\;X\right)-\tau_{\max}\leq 0,s_{2}\left(\;X\right)=\sigma \left(\;X\right)-\sigma_{\max}\leq 0,s_{3}\left(\;X\right)=\delta \left(\;X\right)-\delta_{\max}\leq 0\text{,}$$

$$s_{4}\left(\;X\right)=x_{1}-x_{4}\leq 0,s_{5}\left(\;X\right)=P-P_{c}\left(\;X\right)\leq 0,s_{6}\left(\;X\right)=0.125-x_{1}\leq 0\text{,}$$

$$s_{7}\left(\;X\right)=1.10471x_{1}^{2}+0.04811x_{3}x_{4}\left(14.0+x_{2}\right)-5.0\leq 0$$

The interval for the design variables:

$$0.1\leq x_{1}\leq 2,0.1\leq x_{2}\leq 10,0.1\leq x_{3}\leq 10,0.1\leq x_{4}\leq 2$$

where

$$\tau \left(\;\vec{l}\right)=\sqrt{{\tau^{\prime }}^{2}+2\tau^{\prime }\tau^{\prime \prime \left(\frac{x_{2}}{R}\right)}+{\left(\tau^{\prime \prime }\right)}^{2}},\tau^{\prime }=P/\sqrt{2x_{1}x_{2}},\tau^{\prime \prime }=\text{MR}/J,M=P\left(L+\frac{x_{2}}{2}\right)$$

$$J=2\left\{\sqrt{2}x_{1}x_{2}\left[\left(\frac{\left(x_{2}^{2}\right)}{4}\right)+{\left(x_{1}+\frac{x_{3}}{2}\right)}^{2}\right]\right\},P_{c}\left(\;X\right)=\frac{4.013E\sqrt{\frac{x_{3}^{2}x_{4}^{6}}{36}}}{L^{2}}\left(1-\frac{x_{3}}{2L}\sqrt{E}/4G\;\right)$$

The parameters for WBD are set as follows.$\sigma_{\max}=3000\text{psi},P=6000\;\text{lb},L=14\;\text{in},\delta_{\max}=0.25\text{in},E={3\times 10}^{6}\text{psi},{\;\tau }_{\max}=13600\;\text{psi},\text{and}\;G=12\times 10^{6}\text{psi}$ [86].

In the same vein, the obtained results are presented in Table 13. These results were also compared to the results obtained by analytic means available in the literature shown in Table 14.

**Table 13:** Results for welded beam design problem.

Algorithms	GCRA2	LSHADEcnEpSin	GOA	LSHADE	HHO	LSHADE_SPACMA	WOA	CPSOGSA	RFO [84]	PBO [82]
x1	0.20523	0.247499	0.20573	0.2057	0.205563	0.205396	0.20523	0.20556	0.21846	0.23258
x2	0.20573	0.250284	0.20573	0.2057	0.236204	0.205806	0.20573	0.20581	3.51024	3.49706
x3	3.26225	2.536813	3.47049	3.4705	3.484293	3.4702	3.47049	3.4705	8.87254	8.91026
x4	9.03704	10	9.03662	9.0366	9.03662	9.036624	9.03704	9.03662	0.22491	0.21194
Best	1.6952	−1.23E+06	1.6967	2.55E+22	1.7121	2.55E+22	1.7457	1.7166	1.86612	1.79863
Worst	1.6952	−54931	2.5211	2.55E+22	2.0009	2.55E+22	3.647	2.2837	NA	NA
Average	1.6952	−5.62E+05	1.8894	2.55E+22	1.785	2.55E+22	2.1113	1.8567	NA	NA
Stan.Div	5.68E-07	5.71E+05	0.30372	0	0.064769	0	0.50797	0.13269	NA	NA

**Table 14:** Standard results from classic or analytical approaches in solving welded beam design problems available in the literature [84].

	Method	x1	x2	x3	x4	F(x)*
[87]	Siddall	0.2444	6.2189	8.2915	0.2444	2.38154
[88]	Ragsdell	0.2455	6.1960	8.2730	0.2455	2.38594
[88]	Random	0.4575	4.7313	5.0853	0.6600	4.11856
[88]	Simplex	0.2792	5.6256	7.7512	0.2796	2.53073
[88]	David	0.2434	6.2552	8.2915	0.2444	2.38411
[88]	Approx	0.2444	6.2189	8.2915	0.2444	2.38154

##### 4.2.5.4 Pressure vessel design problem

**Fig. 13:**
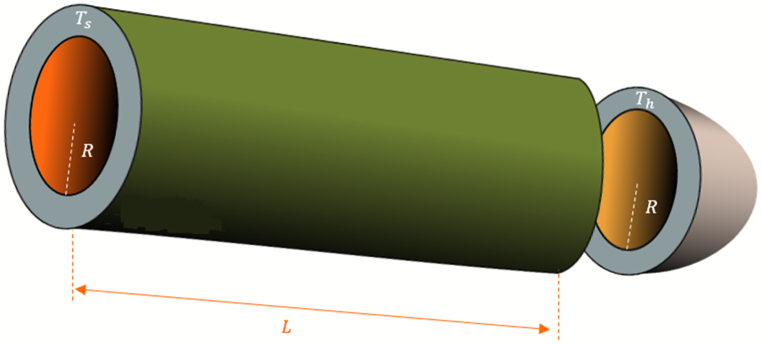
The PVD

The PVD problem pertains to the design of a pressure vessel, as illustrated in Fig. 13. The optimization procedure for PVD is governed by four design variables: the inner radius (R), the head thickness (Tℎ), the length of the vessel's cylindrical section (L), and the shell thickness (Ts). The goal is to reduce the expenses related to raw materials and welding in the construction of the pressure vessel, as depicted in Equation (17).

$$l=\left[T_{s}T_{h}\text{RL}\right]=\left[x_{1},x_{2},x_{3},x_{4}\right]\text{,}$$

17 $$\mathrm{Min}\;f\left(\vec{l}\right)=0.6224x_{1}x_{3}x_{4}1.781x_{2}x_{3}^{2}+3.1661x_{1}^{2}x_{4}+19.84x_{1}^{2}x_{3}$$

$$g_{1}\left(\vec{x}\right)=-x_{1}+0.0193x_{3}\leq 0,g_{2}\left(\vec{x}\right)=-x_{3}+0.00954x_{3}\leq 0,g_{3}\left(\vec{x}\right)=-\pi x_{3}^{2}x_{4}-\frac{4}{3}\pi x_{3}^{3}+1296000\leq 0,g_{4}\left(\vec{x}\right)=x_{4}-240\leq 0\text{.}$$

The design variables specified within the range are listed below.$0\leq x_{1}\leq 99,0\leq x_{2}\leq 99,10\leq x_{3}\leq 200,10\leq x_{4}200$ [85]

Table 15 shows the results of applying candidate metaheuristic optimization to this problem. These results were also compared to the results obtained by analytic means available in the literature shown in Table 16.

**Table 15:** Results for pressure vessel design problem.

Algorithms	GCRA2	LSHADEcnEpSin	GOA	LSHADE	HHO	LSHADE_SPACMA	WOA	CPSOGSA	RFO [84]	PBO [82]
x1	0.446319	0.579194	0.462095	10	10	10	0.69441	0.461506	0.81425	0.81327
x2	0.230237	0.241237	0.240237	10	10	10	0.253932	0.240237	0.44521	0.43702
x3	40.33173	40.37747	40.35339	53.67151	56.07885	53.47238	52.93147	40.32483	42.20231	42.04601
x4	200	200	199.5304	71.64606	56.40497	72.98132	76.66704	200	176.6215	176.756
Best	5367.9	3872.3	4527.3	1.68E+16	4544.1	1.68E+16	4529.4	4530.5	6113.32	6057.547
Worst	15076	5096.1	4527.3	1.68E+16	5579.7	1.68E+16	7539.4	5578.8	NA	NA
Average	5567.7	4923	4527.3	1.68E+16	5230.1	1.68E+16	5615	5050.1	NA	NA
Stan.Div	792.5	424.47	2.82E-10	0	355.28	0	1120.5	369.61	NA	NA

**Table 16:** Standard results from classic or analytical approaches in solving pressure vessel optimization problems available in the literature [84].

	Method	x1	x2	x3	x4	F(x)*
[89]	Lagrange multiplier	1.125	0.625	58.291	43.69	7198.0428
[1]	Branch-bound	1.125	0.625	47.7	117.701	8129.1036

##### 4.2.5.5 Compression spring design problem

**Fig. 14:**
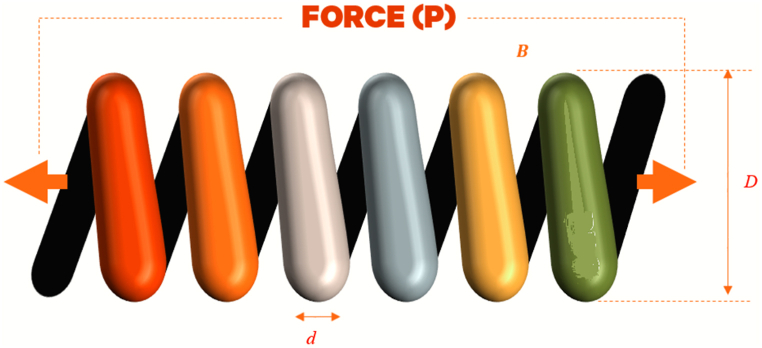
The CSD

The CSD problem shown in Fig. 14, focuses on minimizing the weight of a spring. The spring type considered here is a tension/compression spring. It has three design parameters namely; the wire diameter (d), number of active coils (P), and mean coil diameter (D).

Assume that $l=\left[x_{1}x_{2}x_{3}\right]=\left[\text{dDP}\right]$, the objective function is given in Equation 18

18 $$\mathrm{Min}\;f(\vec{x)}=\left(x_{3}+2\right)x_{2}x_{1}^{2}$$

subject to

$$g_{1}(\vec{x)}=1-\frac{x_{2}^{3}x_{3}}{717851^{4}}\leq 0,g_{2}(\vec{x)}=\frac{4x_{2}^{2}-x_{1}x_{2}}{12566\left(x_{3}x_{1}^{3}-x_{1}^{4}\right)}+1/{5108x_{1}}^{2}\leq 0,g_{3}(\vec{x)}=1-\frac{140.45x_{1}}{x_{2}^{2}x_{3}}\leq 0,g_{4}(\vec{x)}=\frac{x_{1}+x_{2}}{1.5}-1\leq 0\text{.}$$

The intervals for the design variables are.${0.05\leq x}_{1}\leq 2.00,0.25\leq x_{2}\leq 1.30\text{,}$ $2.00\leq x_{3}\leq 15.0$ [90]

The results obtained for GCRA and other metaheuristic algorithms are shown in Table 17. These results were also compared to the results obtained by analytic means available in the literature shown in Table 18.

**Table 17:** Results for compression spring design problem.

Algorithms	GCRA2	LSHADEcnEpSin	GOA	LSHADE	HHO	LSHADE_SPACMA	WOA	CPSOGSA	RFO [84]	PBO [82]
d	0.13915	0.148316747	0.13631	2	2.00008	2	1	0.13785	0.05189	0.05102
D	1.3	1.3	1.22064	1.2	1.2	1.2	1.2	1.26129	0.36142	0.35756
N	11.8924	15	13.2299	2	2	2	3	12.5361	11.58436	11.6994
Best	3.662	−1.48E+09	3.6619	99.986	3.6619	99.986	3.6619	3.6619	0.01321	0.01275
Worst	3.7239	−1.20E+08	3.6619	99.986	3.7311	99.986	3.7313	3.7256	NA	NA
Average	3.6882	−4.33E+08	3.6619	99.986	3.7016	99.986	3.6843	3.6766	NA	NA
Stan.Div	0.02281	4.23E+08	3.95E-16	2.92E-14	0.024346	2.92E-14	0.026358	0.02561	NA	NA

**Table 18:** Standard results from classic or analytical approaches in solving compression spring optimization problems available in the literature [84].

	Method	x1	x2	x3	F(x)*
[91]	Constraint correction	14.25	0.3159	0.05	0.0128334
[92]	Mathematical optimization	9.1854	0.39918	0.053396	0.0127303

##### 4.2.5.6 Cantilever beam design problem

**Fig. 15:**
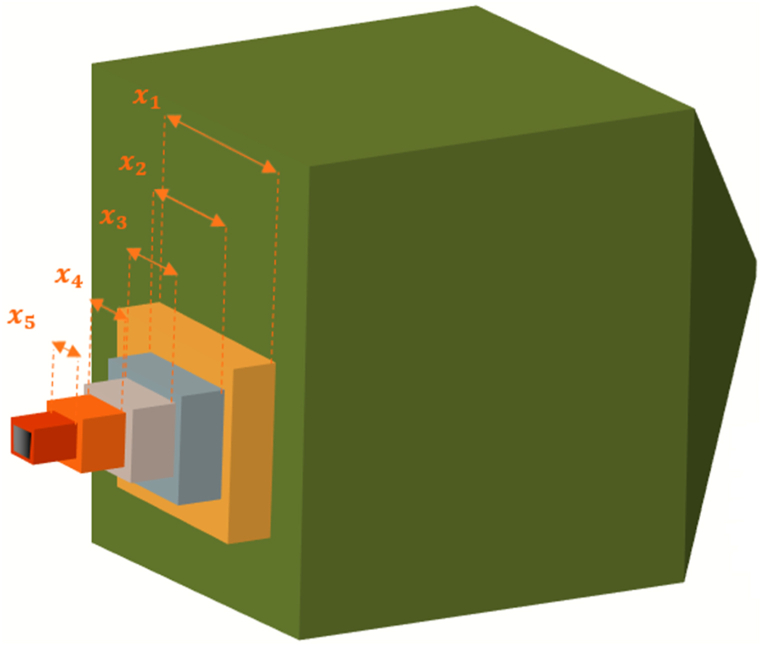
The CBD

Cantilever beams are structural elements that extend freely from one end while being rigidly fixed at the other end as shown in Fig. 15. They find applications in various engineering fields, including civil, mechanical, and aerospace engineering. The goal is to minimize the weight of the cantilever beam, given the decision variables are related to the dimensions of the beam. Equation (19) shows the objective function.

19 $$\min\;f\left(X\right)=0.0624\left(x_{1}+x_{2}+x_{3}+x_{4}+x_{5}\right)$$

Subject to.$g\left(X\right)=\frac{61}{x_{1}^{3}}+\frac{37}{x_{2}^{3}}+\frac{19}{x_{3}^{3}}+\frac{7}{x_{4}^{3}}+\frac{1}{x_{5}^{3}}-1\leq 0\;0.01\leq x_{i}\leq 100\;\forall \;1=1,\ldots ,5$ [78]

Lastly, the results obtained by the metaheuristic algorithms used for this study are presented in Table 19. These results were also compared to the results obtained by analytic means available in the literature shown in Table 20.

**Table 19:** Results for cantilever beam design problem.

Algorithms	GCRA2	LSHADEcnEpSin	GOA	LSHADE	HHO	LSHADE_SPACMA	WOA	CPSOGSA	RFO [84]
x1	5.389588	6.01513	5.689588	5.694297	6.01878	6.01513	5.689588	5.694297	6.00845
x2	5.020725	5.025255	4.41889	5.30344	5.025255	4.41889	5.020755	5.025255	5.30485
x3	4.361692	4.253986	4.119345	4.49587	4.261692	4.253986	4.119345	4.49587	4.49215
x4	3.212994	3.312994	3.314226	7.754706	3.312994	3.314226	7.754706	3.49896	3.49842
x5	2.040863	2.037547	2.15278	2.15428	2.15428	2.040863	2.037547	2.15278	2.14463
Best	1.3705	−35.162	1.3004	1.56E+16	1.3004	1.56E+16	1.3005	1.3004	13.34954
Worst	1.689	−31.235	1.3004	1.56E+16	1.3005	1.56E+16	1.3011	1.3004	NA
Average	1.5127	−31.935	1.3004	1.56E+16	1.3004	1.56E+16	1.3006	1.3004	NA
Stan.Div	0.09707	0.9136	3.14E-16	2.052	6.74E-06	2.052	0.000195	3.71E-16	NA

**Table 20:** Standard results from classic or analytical approaches in solving cantilever beam optimization problems available in the literature [84].

	Method	x1	x2	x3	x4	x5	F(x)*
[93]	Generalized Convex Approximation	6.01	5.3	4.49	3.49	2.15	13.3442
[93]	Moving Asymptotes	6.01	5.3	4.49	3.49	2.15	13.3442

#### 4.2.6 Results discussion of the optimization of engineering problems

For the tree bar truss problem, the GCRA, GOA, HHO, and WOA found the lowest value of the cost function at least once. Throughout 30 runs of the algorithms, the GOA returned the lowest average value of the cost function closely followed by the proposed GCRA and red fox optimization algorithm (RFO). We noticed that the LSHADE and its two variants used in this study returned abnormal results. This can be attributed to the parameter used, with better fine-tuning, we believe they will leave to their potential. Considering the welded beam problem, similar observations can be seen, with GCRA returning the lowest average value of the cost function over 30 runs. The GOA was very competitive too, closely following the GCRA. The RFO and polar bear optimization algorithm (PBO) were placed 5th and 6th respectively. Comparing these results to those of Table 14 (literature results), it can be seen that they are close to those of Siddall, Ragsdell, and David's methods.

Furthermore, in the compression spring problem, the RFO and PBO returned results close to the standard results from classic/analytical approaches in the literature shown in Table 18. It can observed that the GCRA, GOA, HHO, WOA, and CPSOGSA returned far above the lowest in Table 18, however, the GCRA closely followed the RFO and PBO. For the cantilever beam design problem, the GCRA, GOA, HHO, WOA, and CPSOGSA returned results far below the RFO and those in Table 20. Looking further, it can be noticed that the proposed GCRA closely followed the RFO, which is an advantage for the GCRA as a promising algorithm.

In the pressure vessel problem, the GOA found the minimum value of the cost function, with the GCRA trailing closely behind. However, the RFO's result is more aligned with the results in Table 16, demonstrating the GCRA's competitiveness against other methods. In general, the techniques used in this problem achieved lower cost function values than the Lagrange multiplier and branch-bound methods presented. For the gear train problem, the RFO returned the best result, followed closely by the GCRA and GOA. The top results were obtained by the RFO and GCRA, with a negligible difference between them. It's important to note that the results obtained from all heuristics vary significantly in general.

These experiments demonstrate the competitiveness of the proposed GCRA, consistently ranking among the top two in all engineering problems tackled in this study. While it may not be the absolute best among the evaluated heuristics, it's noteworthy that the GCRA performs comparably to renowned classical algorithms in a similar number of experiments, which is an impressive outcome.

### 4.3 Statistical analysis

The results in Section 4.2 provide significant statistics that offer insights into the exploration and exploitation capabilities of the proposed algorithm. However, these alone are insufficient to validate the algorithm's effectiveness. Additional statistical tests using 'mean' values are required to demonstrate the robustness and efficacy of the proposed algorithm. Both Friedman's and Wilcoxon's signed-rank tests were employed for further analysis of the results.

**Fig. 16:**
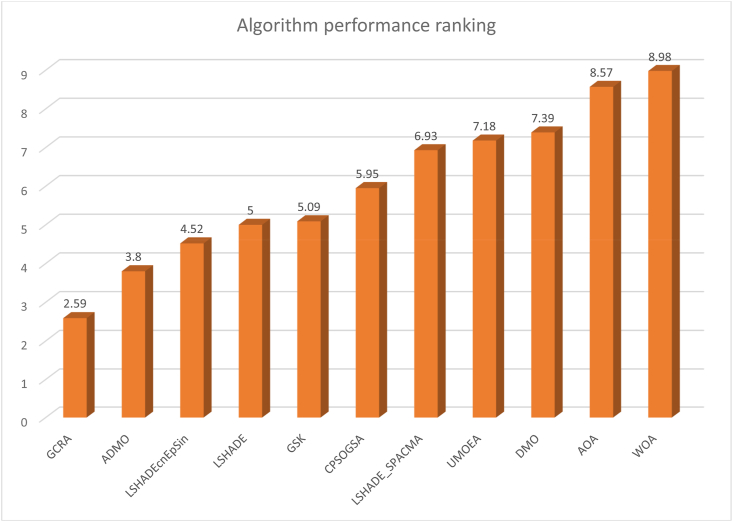
Graphical representation of algorithm's performance ranking (algorithm with the least value is ranked higher).

The outcomes of Friedman's test are displayed in Table 21. In the context of Friedman's test, a lower mean rank indicates superior performance. Furthermore, Friedman's test presumes that "the mean distribution of the obtained result is identical." The significance level is typically set at α = 0.05. As per Table 8, the p-value = 0.000 is less than α, leading to the rejection of the hypothesis. The GCRA ranks first as it has the lowest mean rank. The ADMO follows closely behind the GCRA, then the LSHADEcnEpSin, and LSHADE. Fig. 16 illustrates the performance ranking.

**Table 21:** Friedman's test results.

Algorithm	Mean Rank	Ranking
GCRA	2.59	1
ADMO	3.8	2
LSHADEcnEpSin	4.52	3
LSHADE	5	4
GSK	5.09	5
CPSOGSA	5.95	6
LSHADE_SPACMA	6.93	7
UMOEA	7.18	8
DMO	7.39	9
AOA	8.57	10
WOA	5.09	11
Test Statistics^a^		
N	22	
Chi-Square	84.486	
df	10	
Asymp. Sig.	<,001

Following Friedman's test, Wilcoxon's test was conducted to provide a pairwise performance comparison between GCRA and the other algorithms. The findings of this test are summarized in Table 22. The results indicate that the GCRA significantly outperforms all the algorithms except the LSHADEcnEpSin and GSK across all considered problems. This conclusion is based on the high R+ values achieved by the GCRA. Specifically, at α = 0.05, the GCRA significantly outperformed 8 out of the 10 algorithms. These results further affirm the GCRA's performance in terms of searchability, stability, and efficiency in solving CEC 2011 real-world optimization problems.

**Table 22:** Comparative results of Wilcoxon's test.

Algorithm		N	Mean Rank	Sum of Ranks	P-value	Asymp. Sig. (2-tailed)
ADMO - GCRA	Negative Ranks	1	1.00	1.00	−2.982b	0.003
	Positive Ranks	11	7.00	77.00		
	Ties	10				
	Total	22				
LSHADEcnEpSin - GCRA	Negative Ranks	3	10.67	32.00	−2.107^b^	0.035
	Positive Ranks	14	8.64	121.00		
	Ties	5				
	Total	22				
LSHADE - GCRA	Negative Ranks	3	6.00	18.00	−3.248^b^	0.001
	Positive Ranks	17	11.29	192.00		
	Ties	2				
	Total	22				
DMO - GCRA	Negative Ranks	1	6.00	6.00	−3.912^b^	<,001
	Positive Ranks	21	11.76	247.00		
	Ties	0				
	Total	22				
LSHADE_SPACMA - GCRA	Negative Ranks	3	5.83	17.50	−3.273^b^	0.001
	Positive Ranks	17	11.32	192.50		
	Ties	2				
	Total	22				
UMOEA - GCRA	Negative Ranks	3	3.00	9.00	−3.461^b^	<,001
	Positive Ranks	16	11.31	181.00		
	Ties	3				
	Total	22				
WOA - GCRA	Negative Ranks	1	4.00	4.00	−3.977^b^	<,001
	Positive Ranks	21	11.86	249.00		
	Ties	0				
	Total	22				
AOA - GCRA	Negative Ranks	1	4.00	4.00	−3.977^b^	<,001
	Positive Ranks	21	11.86	249.00		
	Ties	0				
	Total	22				
CPSOGSA - GCRA	Negative Ranks	2	4.50	9.00	−3.702^b^	<,001
	Positive Ranks	19	11.68	222.00		
	Ties	1				
	Total	22				
GSK - GCRA	Negative Ranks	3	9.00	27.00	−2.343^b^	0.019
	Positive Ranks	14	9.00	126.00		
	Ties	5				
	Total	22				

b . Based on negative ranks.

### 4.4 Summary of results

The six engineering optimization problems and the CEC 2011 real-world test suite, which encompasses a broad spectrum of optimization problems, were utilized to evaluate the robustness and effectiveness of the GCRA. The experimental outcomes of the proposed algorithm were juxtaposed with ten other cutting-edge algorithms. These algorithms include the LSHADE, LSHADEcnEpSin, LSHADE_SPACMA, and UMOEA, which are the four high-performance algorithms from CEC competitions. Also, the GSK, a human-based algorithm, was used too. Finally, three swarm-based algorithm representations (AOA, CPSOGSA, WOA) were used in this study. Further analysis of the obtained results was conducted using two statistical methods, namely Friedman's and Wilcoxon's tests.

The proposed GCRA demonstrated highly competitive performance, finding the optimal solution for most of the optimization functions used in this study. This performance can be attributed to the effective foraging method employed by the GCR during and outside the mating season, as meticulously modeled in Equations 4, 5, 8, 9. According to Friedman's test, the GCRA ranked first, closely followed by LSHADE, LSHADEcnEpSin, LSHADE_SPACMA, GSK, and UMOEA. Further results from Wilcoxon's signed ranks test confirmed the GCRA's superiority and competitiveness compared to the other algorithms used in this study across all functions in the test suite.

In terms of convergence analysis, the GCRA demonstrated rapid and steady convergence in the initial few iterations for most optimization problems defined in CEC 2011, except for F1 and F3, which exhibited delayed convergence, stabilizing only in the later stages of the iterations. It was observed that the high-performing algorithms were stable around the global or near-global solutions, necessitating few iterations for most functions.

## 5 Conclusion and future work

Greater cane rats (GCR) exhibit territorial behavior, with instances of male-only conflicts using their noses for combat. They are social creatures that live in groups consisting of a dominant male, multiple females, and young ones that can be more than one generation old. The GCR's foraging behavior involves cutting canes and grasses with their specially adapted upper incisors. As highly nocturnal animals, they are smart enough to leave trails while foraging through reeds and grass, leading to food, water, and shelter. This intelligent behavior of the GCR is the main inspiration for this study. The exploration phase is realized when they leave various shelters within their territory to forage and create trails.

It is assumed that the dominant male retains the information about these trails and other rats subsequently update their position based on this data. Males can also recognize the breeding season and isolate themselves from the group. It is assumed that once the group separates during this season, foraging activities are focused in areas with plentiful food sources, facilitating exploitation. Hence, the intelligent foraging trails and actions during the mating season are mathematically modeled to design the GCR algorithm and carry out optimization activities.

The CEC 2011 real-world test suite, which includes a broad spectrum of optimization problems, was used to evaluate the effectiveness and robustness of GCRA. The experimental results of the proposed algorithm were compared with 10 other cutting-edge algorithms. The performance of the proposed GCRA was highly competitive, finding the optimal solution in most of the optimization functions used in this study. This performance can be attributed to the efficient foraging method used by the GCR during and outside the mating season, meticulously modeled in Equations 4, 5, 8, 9. Friedman's test revealed that GCRA ranked first, closely followed by LSHADE, LSHADEcnEpSin, LSHADE_SPACMA, GSK, and UMOEA. Further results from Wilcoxon's signed ranks test confirmed GCRA's superiority and competitiveness compared with other algorithms used in this study for all functions in the test suite.

Also, in the experiments for the six (6) engineering problems, we see how competitive the proposed GCRA is, always within the best 2 in all engineering problems solved in this study. So in general while it cannot be said that the proposed algorithm was the best algorithm among examined heuristics, it can be said that the GCRA succeeds in a similar number of experiments as well-known classical algorithms, which is a very good result.

The proposed GCRA is straightforward to implement and has demonstrated its reliability, efficiency, and robustness in real parameter optimization. The current GCRA design has proven effective in resolving the CEC 2011 real-world test functions and six engineering design problems. Future work will aim to modify the GCRA to make it suitable for solving other constrained multi-objective optimization problems, both discrete and continuous, and other practical optimization problems in fields like engineering and scheduling. Another promising area of research will be the integration of machine learning and artificial intelligence into individual GCRs to facilitate their evolution in subsequent generations. A comprehensive theoretical and parametric study of the GCRA is another beneficial direction. Lastly, hybridization might be a valuable strategy for enhancing the performance of the GCRA.

## Ethical Approval

NA.

## Availability of data and materials

All data generated or analyzed during this study are included in this article.

## CRediT authorship contribution statement

**Jeffrey O. Agushaka:** Conceptualization. **Absalom E. Ezugwu:** Conceptualization. **Apu K. Saha:** Conceptualization. **Jayanta Pal:** Conceptualization. **Laith Abualigah:** Conceptualization. **Seyedali Mirjalili:** Conceptualization.

## Declaration of competing interest

The authors declare that they have no known competing financial interests or personal relationships that could have appeared to influence the work reported in this paper.

## Figures and Tables

**Table A1:** Classical benchmark functions

ID	Type	Function	Dimension	Bounds	Global
F1	Unimodal	$f\left(x\right)=\sum_{i=1}^{n}x_{i}^{2}$	30	[-100,100]	0
F2	Unimodal	$f\left(x\right)=\sum_{i=0}^{n}\left|x_{i}\right|+\prod_{i=0}^{n}\left|x_{i}\right|$	30	[-10,10]	0
F3	Unimodal	$f\left(x\right)=\sum_{i=1}^{d}{\left(\sum_{j=1}^{i}x_{j}\right)}^{2}$	30	[-100,100]	0
F4	Unimodal	$f\left(x\right)=\max_{i}\{\left|x_{i}\right|,1\leq i\leq n\}$	30	[-100,100]	0
F5	Unimodal	$f\left(x\right)=\sum_{i=1}^{n-1}{[100\left(x_{i}-x_{i+1}\right)}^{2}+{\left(1-x_{i}\right)}^{2}]$	30	[-30,30]	0
F6	Unimodal	$f\left(x\right)=\sum_{i=1}^{n}{\left(\left[x_{i}-0.5\right]\right)}^{2}$	30	[-100,100]	0
F7	Unimodal	$f\left(x\right)=\sum_{i=0}^{n}ix_{1}^{4}+\text{rand}[0,1)$	30	[-128,128]	0
F8	Multimodal	$f\left(x\right)=\sum_{i=1}^{n}-x_{i}\;\sin\left(\sqrt{\left|x_{i}\right|}\right)$	30	[-500,500]	-418.9829×5
F9	Multimodal	$f\left(x\right)=10+\sum_{i=1}^{n}{(x}_{i}^{2}-10\;\cos\;\left(2\pi x_{i}\right))$	30	[-5.12,5.12]	0
F10	Multimodal	$f\left(x\right)=-a\;\exp\left(-0.02\sqrt{n^{-1}\sum_{i=1}^{n}x_{i}^{2}}\right)-\exp\left(n^{-1}\sum_{i=1}^{n}\cos\left(2\pi x_{i}\right)\right)+a+e,a=20$	30	[-32,32]	0
F11	Multimodal	$f\left(X\right)=1+\frac{1}{4000}\sum_{i=1}^{n}x_{i}^{2}-\prod_{i=1}^{n}\cos\left(\frac{x_{i}}{\sqrt{i}}\right)$	30	[-600,600]	0
F12	Multimodal	$f\left(x\right)=\frac{\pi }{n}\left\{10\;\sin\left(\pi y_{i}\right)\right\}+\sum_{i=1}^{n-1}{\left(y_{i}-1\right)}^{2}\left[1+10\;\sin^{2}\left(\pi y_{i+1}\right)+\sum_{i=1}^{n}u\left(x_{i},10,100,4\right)\right]$ Where $y_{i}=1+\frac{x_{i}+1}{4},u\left(x_{i},a,k,m\right)\left\{ \begin{array}{c} K{\left(x_{i}-a\right)}^{m}\;\text{if}\;x_{i}>a\\ 0\;-a\leq x_{i}\geq a\;\\ K{\left(-x_{i}-a\right)}^{m}\;-a\leq x_{i} \end{array}\right.$	30	[-50,50]	0
F13	Multimodal	$f\left(x\right)=0.1(\sin^{2}\left(3\pi x_{1}\right)+\sum_{i=1}^{n}{\left(x_{i}-1\right)}^{2}\left[1+\sin^{2}\left(3\pi x_{i}+1\right)\right]+{{\left(x_{n}-1\right)}^{2}1+\sin}^{2}\left(2\pi x_{n}\right))+\sum_{i=1}^{n}u\left(x_{i},5,100,4\right)$	30	[-50,50]	0
F14	Fixed-dimension multimodal	$f\left(x\right)={\left(\frac{1}{500}+\sum_{j=1}^{25}\frac{1}{j+\sum_{i=1}^{2}{\left(x_{i}-a_{\text{ij}}\right)}^{6}}\right)}^{-1}$	2	[-65,65]	1
F15	Fixed-dimension multimodal	$f\left(x\right)=\sum_{i=1}^{11}{\left[a_{i}-\frac{x_{1}\left(b_{i}^{2}+b_{i}x_{2}\right)}{b_{i}^{2}+b_{i}x_{3}+x_{4}}\right]}^{2}$	4	[-5,5]	0.00030
F16	Fixed-dimension multimodal	$f\left(x\right)=ax_{1}^{2}-2.1x_{1}^{4}+\frac{1}{3}x_{1}^{6}+x_{1}x_{2}-4x_{2}^{2}+4x_{2}^{4}$	2	[-5,5]	−1.0316
F17	Fixed-dimension multimodal	$f\left(X\right)=\left[1+{\left(x_{1}+x_{2}+1\right)}^{2}\left(19-14x_{1}+3{x_{1}}^{2}-14x_{2}+6x_{1}x_{2}+3{x_{2}}^{2}\right)\right]\times \left[30+{\left(2x_{1}-3x_{2}\right)}^{2}(18-32x_{1}+12{x_{1}}^{2}+48x_{2}-36x_{1}x_{2}+27{x_{2}}^{2}\right]$	2	[-2,2]	3
F18	Fixed-dimension multimodal	$f\left(x\right)=-\sum_{i=1}^{4}c_{i}\;\exp\left(-\sum_{i=1}^{3}a_{\text{ij}}{\left(x_{j}-p_{\text{ij}}\right)}^{2}\right)$	3	[-1,2]	−3.86
F19	Fixed-dimension multimodal	$f\left(x\right)=-\sum_{i=1}^{4}c_{i}\;\exp\left(-\sum_{i=1}^{6}a_{\text{ij}}{\left(x_{j}-p_{\text{ij}}\right)}^{2}\right)$	6	[0,1]	−3.2
F20	Fixed-dimension multimodal	$f\left(x\right)=-\sum_{i=1}^{5}{\left[\left(X-a_{i}\right){\left(X-a_{i}\right)}^{T}+c_{i}\right]}^{-1}$	4	[0,1]	−10.1532
F21	Fixed-dimension multimodal	$f\left(x\right)=-\sum_{i=1}^{7}{\left[\left(X-a_{i}\right){\left(X-a_{i}\right)}^{T}+c_{i}\right]}^{-1}$	4	[0,1]	−10.4028
F22	Fixed-dimension multimodal	$f\left(x\right)=-\sum_{i=1}^{10}{\left[\left(X-a_{i}\right){\left(X-a_{i}\right)}^{T}+c_{i}\right]}^{-1}$	4	[0,1]	−10.5363

**Table A2:** CEC 2020

Type	Number	Functions	$F_{i}^{*}$
**Unimodal** **Function**	1	Shifted and Rotated Bent Cigar Function (CEC 2017 [4] F1)	100
**Basic** **Functions**	2	Shifted and Rotated Schwefel's Function (CEC 2014 [3] F11)	1100
	3	Shifted and Rotated Lunacek bi-Rastrigin Function (CEC 2017 [4] F7)	700
	4	Expanded Rosenbrock's plus Griewangk's Function (CEC2017 [4] f19)	1900
**Hybrid** **Functions**	5	Hybrid Function 1 (N = 3) (CEC 2014 [3] F17)	1700
	6	Hybrid Function 2 (N = 4) (CEC 2017 [4] F16)	1600
	7	Hybrid Function 3 (N = 5) (CEC 2014 [3] F21)	2100
**Composition** **Functions**	8	Composition Function 1 (N = 3) (CEC 2017 [4] F22)	2200
	9	Composition Function 2 (N = 4) (CEC 2017 [4] F24)	2400
	10	Composition Function 3 (N = 5) (CEC 2017 [4] F25)	2500

* Search range: [-100,100]^D^.
